# Transcriptome analysis reveals new insight into appressorium formation and function in the rice blast fungus *Magnaporthe oryzae*

**DOI:** 10.1186/gb-2008-9-5-r85

**Published:** 2008-05-20

**Authors:** Yeonyee Oh, Nicole Donofrio, Huaqin Pan, Sean Coughlan, Douglas E Brown, Shaowu Meng, Thomas Mitchell, Ralph A Dean

**Affiliations:** 1 North Carolina State University, Center for Integrated Fungal Research, Raleigh, NC 27695-7251, USA; 2 Agilent Technologies, Little Falls, DE 19808-1644, USA; 3 Current address: University of Delaware, Department of Plant and Soil Science, Newark, DE 19716, USA; 4 Current address: RTI international, Research Triangle Park, NC 27709-2194, USA; 5 Current address: Ohio State University, Department of Plant Pathology, Columbus, OH 43210, USA

## Abstract

**Background:**

Rice blast disease is caused by the filamentous Ascomycetous fungus *Magnaporthe oryzae *and results in significant annual rice yield losses worldwide. Infection by this and many other fungal plant pathogens requires the development of a specialized infection cell called an appressorium. The molecular processes regulating appressorium formation are incompletely understood.

**Results:**

We analyzed genome-wide gene expression changes during spore germination and appressorium formation on a hydrophobic surface compared to induction by cAMP. During spore germination, 2,154 (approximately 21%) genes showed differential expression, with the majority being up-regulated. During appressorium formation, 357 genes were differentially expressed in response to both stimuli. These genes, which we refer to as appressorium consensus genes, were functionally grouped into Gene Ontology categories. Overall, we found a significant decrease in expression of genes involved in protein synthesis. Conversely, expression of genes associated with protein and amino acid degradation, lipid metabolism, secondary metabolism and cellular transportation exhibited a dramatic increase. We functionally characterized several differentially regulated genes, including a subtilisin protease (*SPM1*) and a NAD specific glutamate dehydrogenase (*Mgd1*), by targeted gene disruption. These studies revealed hitherto unknown findings that protein degradation and amino acid metabolism are essential for appressorium formation and subsequent infection.

**Conclusion:**

We present the first comprehensive genome-wide transcript profile study and functional analysis of infection structure formation by a fungal plant pathogen. Our data provide novel insight into the underlying molecular mechanisms that will directly benefit efforts to identify fungal pathogenicity factors and aid the development of new disease management strategies.

## Abstract 2

Analysis of genome-wide gene-expression changes during spore germination and appressorium formation in *Magnaporthe oryzae* revealed that protein degradation and amino-acid metabolism are essential for appressorium formation and subsequent infection.

## Background

In the course of evolution, organisms have adapted to exploit diverse habitats, including the ability to grow and reproduce at the expense of others. Many pathogens have evolved sophisticated strategies to first attach to and subsequently infect their hosts, processes that often involve unique morphological changes. Discovery of the underlying molecular mechanisms of how pathogens first recognize hosts and set in motion the infection process is not only central to understanding pathogen biology, but requisite for the development of effective disease control strategies. The perception of cues from a host typically trigger a cascade of cellular processes whereby a signal is relayed from the cell surface to the nucleus, resulting in activation of gene expression and, in the case of many fungal pathogens, specific developmental changes. *Magnaporthe oryzae *is typical of many fungal pathogens of plants in that it elaborates a specialized infection cell called an appressorium to infect its host. *M. oryzae *is the causal agent of rice blast, the most destructive fungal disease of rice worldwide and a seminal model for the study of the molecular basis of fungal-plant interactions. It was the first filamentous fungal pathogen to have a complete genome sequence publicly available [1].

Following spore attachment and germination on the host surface, an emerging germ tube perceives physical cues, such as surface hardness and hydrophobicity, as well as chemical signals, including wax monomers, that trigger appressorium formation [2-4]. Appressorium formation begins when the tip of the germ tube ceases polar growth, hooks, and begins to swell. The contents of the spore are then mobilized into the developing appressorium, a septum develops at the neck of the appressorium, and the germ tube and spore collapse and die. As the appressorium matures, it becomes firmly attached to the plant surface and a dense layer of melanin is laid down in the appressorium wall, except across a pore at the plant interface. Turgor pressure increases inside the appressorium and a penetration hyphae emerges at the pore, which is driven through the plant cuticle into the underlying epidermal cells [5-10]. Melanin deposition in the cell wall of the appressorium is essential for maintaining turgor pressure. Genetic mutations or chemical treatments that inhibit appressorium formation and function effectively block penetration and subsequent disease development [7,11].

Highly conserved signaling networks that transfer cues from the environment to the nucleus play a crucial role in regulating pathogen-host interactions. For *M. oryzae*, the mitogen-activated protein kinase (MAPK), cyclic AMP (cAMP) and to a lesser extent Ca^2+ ^signaling pathways have been shown to be essential for appressorium formation and function [12-16]. In addition, the cAMP signaling pathway regulates several other aspects of fungal growth and development, including nutrient sensing and cell morphogenesis [17-19]. In *M. oryzae*, exogenous cAMP and analogs induce appressorium formation in non-inductive environments [20]. Subsequent functional characterization of genes encoding proteins in the cAMP signaling pathway, including *MagB*, alpha subunit of G protein, *Mac1*, adenylyl cyclase, and *cPKA*, the catalytic subunit of protein kinase A, provided clear evidence for the essential role of cAMP in regulating appressorium morphogenesis [13,21-23]. These pioneering studies served as the catalyst to drive numerous studies in other pathogenic fungi such as *Blumeria*, *Colletotricum*, *Fusarium*, and *Sclerotinia *species [24-27]. However, while the core pathways are highly conserved, relatively little is known of the downstream genes and pathways that direct infection related morphogenesis.

Appressorium function is dependent on generating high levels of turgor, which in *M. oryzae *results from high concentrations of glycerol. How glycerol is generated in the appressoria remains to be clearly defined, but because appressoria develop in the absence of nutrients, it has been suggested that glycerol must be derived from storage products. Carbohydrate catabolism in yeast is regulated by the cAMP response pathway; however, there is no genetic evidence that metabolism of storage glycogen or trehalose is required for appressorium turgor generation [28]. *TRE1*, which encodes the main intracellular trehalase activity in spores, is not required for appressorium function [29]. On the other hand, targeted mutagenesis of genes involved in degradation of storage lipids or beta oxidation of fatty acids, such as *MFP1*, or genes involved in peroxisome function, such as *MgPEX6*, prevent appressorium function but do not appear to affect the accumulation of glycerol [30]. Thus, although spores and developing appressoria contain substantial amounts of lipids and carbohydrates, it appears that glycerol may be derived from other cellular materials. Appressorium formation is accompanied by collapse of the spore, a process involving autophagy whereby cellular contents of the spore are re-cycled into the developing appressorium. Autophagy genes *MgATG1 *or *MgATG8 *are required for normal appressorium formation and deletion mutants are non-pathogenic [31,32]. This opens up the possibility that glycerol may be derived from materials other than lipids and carbohydrates.

Studies of appressorium formation and early stages of host invasion suggest that *M. oryzae *is not only capable of perceiving its host but is able to evade host detection during pre-penetration and tissue colonization [33]. Bacteria have evolved a specialized type III secretion system to deliver proteins into plant cells to help evade host recognition and promote invasive growth [34]. *M. oryzae *mutants defective in secretion, *MgAPT2 *deletion strains, for example, are unable to cause disease [35]. Thus, secreted proteins likely play a significant role in fungal pathogenesis. The *M. oryzae *genome contains a large and diverse complement of secreted proteins; however, their function remains largely unknown. Other effector molecules, including secondary metabolites, may be delivered by transporters. It is known that ATP-binding cassette (ABC) type transporters such as *ABC3 *are required for appressorium function [36]. *M. oryzae *contains at least 23 polyketide synthases, several non-ribosomal peptide synthases and more than 120 highly diverged cytochrome P450 monooxygenases, suggesting a significant capacity to produce a diverse array of secondary metabolites [1]. The nature of these metabolites and the role they play in the infection process is not well defined.

Although evidence collected to date, primarily from studies of *M. oryzae*, provides important clues as to processes involved in appressorium formation and function, a complete understanding of the metabolic changes and genes contributing to infection related morphogenesis is far from complete. One powerful method for refining and extending knowledge of the infection process is to identify alterations in transcription as *M. oryzae *undergoes appressorium formation. To date, very limited gene expression studies have been performed to identify genes associated with appressorium formation and function in fungal pathogens [37-43]. Published studies have examined only small subsets of the total gene complement from fungal pathogens and have been far from exhaustive. The recent completion of the *M. oryzae *genome sequence greatly enables genomic analyses [1].

**Figure 1:**
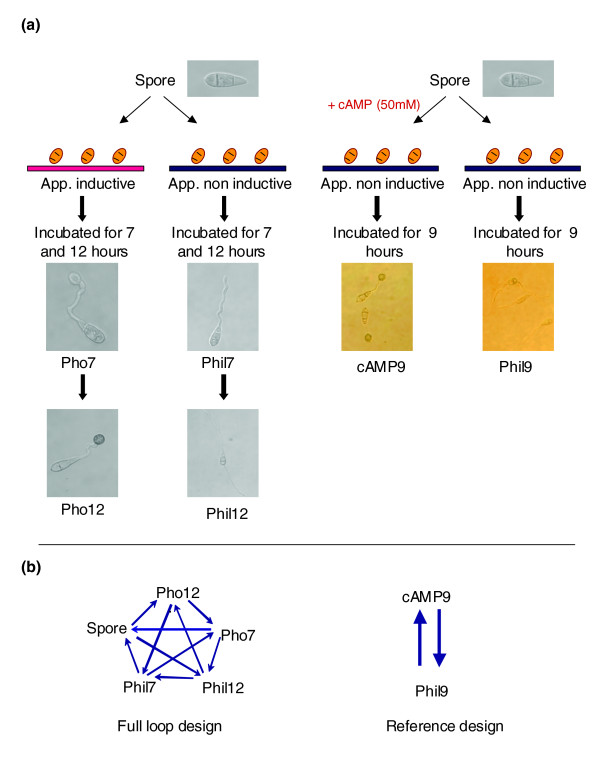
Experimental and microarray design for spore germination and appressorium induction. **(a) **Spores were placed on the hydrophilic (Phil) and hydrophobic (Pho) surfaces of GelBond and incubated for 7 and 12 h. For induction of appressoria by cAMP, spores were placed on the hydrophilic surface of GelBond with (cAMP9) and without (Phil9) cAMP and incubated for 9 h. **(b) **Diagrams show microarray design. Arrows connect samples directly compared on two channel Agilent *M. oryzae *oligonucleotide microarrays. Arrow heads = Cy5, arrow tails = Cy3.

In this study, we made use of a whole genome oligo microarray chip containing over 13,000 *M. oryzae *elements representing 10,176 predicted genes, and conducted global gene expression profiles during spore germination and appressorium formation on both an inductive hydrophobic surface and in response to cAMP (Figure 1). From these data, we distilled a consensus set of genes differentially expressed in response to both physical and chemical cues, and constructed putative biological pathways that participate in appressorium formation. Our data show that germination stimulates a major transcriptional response characterized by a dramatic increase in expression of genes involved in metabolism and biosynthesis. On the other hand, induction of appressorium formation triggers a significant decrease in expression of genes associated with the translational apparatus, with a coordinate increase in the expression of genes involved in protein and amino acid degradation, lipid metabolism, secondary metabolism and cellular transportation. Significantly, the set of up-regulated genes is enriched for those encoding predicted secreted proteins. To directly assay the role of these gene sets in appressorium formation and function, we performed targeted gene deletion studies on many of the most highly up-regulated genes. Our findings reveal that protein degradation and amino acid metabolism are essential for the infection process. Further, we find many differentially expressed genes are not required for appressorium formation and function. This may suggest that *M. oryzae *employs a number of backup systems, such as functional redundancy and compensatory processes in order to protect appressorium formation from being de-regulated.

## Results

### Genes involved in core biological processes undergo dramatic transcriptional changes during spore germination

Microarray analysis revealed that about 29% of the 10,176 *M. oryzae *genes present on the array underwent significant changes (≥ 2-fold, *p *< 0.05) in expression during at least one of the developmental processes tested, including spore germination, germ tube elongation or appressorium development (Table 1). The most dramatic change in gene expression occurred during spore germination (Phil7 versus Spore) where approximately 21% showed differential expression with the vast majority being up-regulated. Seventy three percent of the genes differentially expressed during spore germination exhibited no further change in expression during germ tube elongation or appressorium formation. Very few further changes (<1%) in gene expression were observed during germ tube elongation (Phil12 versus Phil7).

**Table 1:** Differential expression of 10,176 *M. oryzae *genes during spore germination and appressorium formation

	SG_up*	SG_dn	GE_up	GE_dn	AI_up	AI_dn	AM_up	AM_dn	CI_up	CI_dn
SG_up	2,087^†^									
SG_dn		67								
GE_up	17	2	46							
GE_dn	13			17						
AI_up	62	2			178					
AI_dn	107	2	3	11		142				
AM_up	50	7	2		84	2	267			
AM_dn	128	5	19	4	1	71		179		
CI_up	113	8	7	2	103	2	202	1	644	
CI_dn	242	10	13	7		71		92		370

*Abbreviations indicate particular expression profiles: SG, spore germination; GE, germ tube elongation; AI, appressorium initiation; AM, appressorium maturation; CI, cAMP induced appressorium. Up, up-regulated; dn, down-regulated. ^†^Each value indicates the number of genes in common between the paired expression profiles.

To explore the cellular processes active during spore germination, differentially expressed genes were first grouped according to Gene Ontology (GO) terms. Examination of gene expression with GO categories revealed that during spore germination, genes involved in major biological processes such as metabolism (GO:0008152) and biosynthesis (GO:0009058) were significantly over-represented (*p *< 0.01) in the up-regulated gene set (Additional data file 1). In particular, genes associated with carbohydrate metabolism (GO:0005975), amino acid and derivative metabolism (GO:0006519) and protein metabolism (GO:0019538) were over-represented. In contrast, genes associated with the GO category for transcription (GO:0006350) were under-represented in the up-regulated gene set. The GO category for transcription contains mainly transcription factors and other proteins involved in DNA binding. Typically, transcription factors are post transcriptionally regulated and, thus, their expression would not necessarily be expected to be over-represented during spore germination (Additional data file 1).

### Thigmotrophic and chemical induction of appressorium formation trigger similar patterns of gene expression

**Figure 2:**
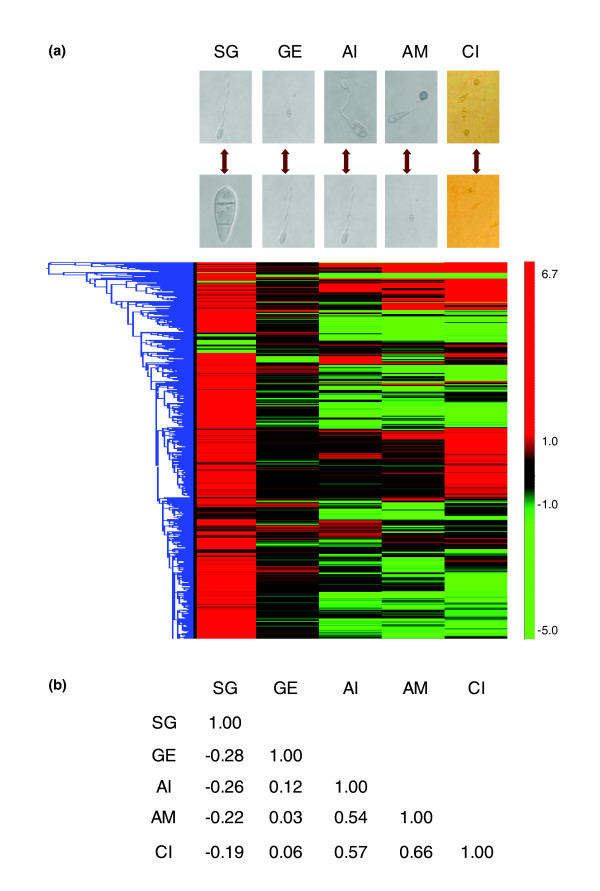
Gene expression profile clustering and correlation analysis. **(a) **Hierarchical clustering analysis of gene expression profiless for spore germination (SG), germ tube elongation (GE), appressorium initiation (AI), appressorium maturation (AM) and cAMP-induced appressoria (CI). Differential expression of each gene is indicated in color (red shows induced, green shows repressed, and numbers next to scale indicate fold change (log2)). **(b) **Correlation coefficient for pairwise gene expression profiles shown in (a).

Approximately 3-4% of the entire set of *M. oryzae *open reading frames were differentially expressed during appressorium initiation (Pho7 versus Phil7) and maturation (Pho12 versus Phil12) on the inductive surface. In response to exogenous cAMP, about 10% of expressed genes were differentially expressed (cAMP9 versus Phil9; Table 1). Considerably more genes were found to be induced rather than repressed by both physical and chemical (cAMP) stimulation. Overall, good correlations (Pearson's correlation coefficient r > 0.5) were observed between appressorium related expression profiles induced by physical cues (appressorium initiation and maturation) and by cAMP (Figure 2). In contrast, gene expression profiles during spore germination and germ tube elongation correlated poorly with those observed for appressorium formation. The highest correlation (r = 0.66) was found between appressorium maturation and cAMP induced appressoria where 66% of differentially expressed genes showed a similar expression pattern. Approximately 54% of genes differentially expressed during appressorium initiation exhibited a similar expression pattern in response to cAMP (Table 1, Figure 2).

### Microarray based gene expression pattern is consistent with expression analysis from reverse transcriptase PCR and quantitative RT-PCR

**Figure 3:**
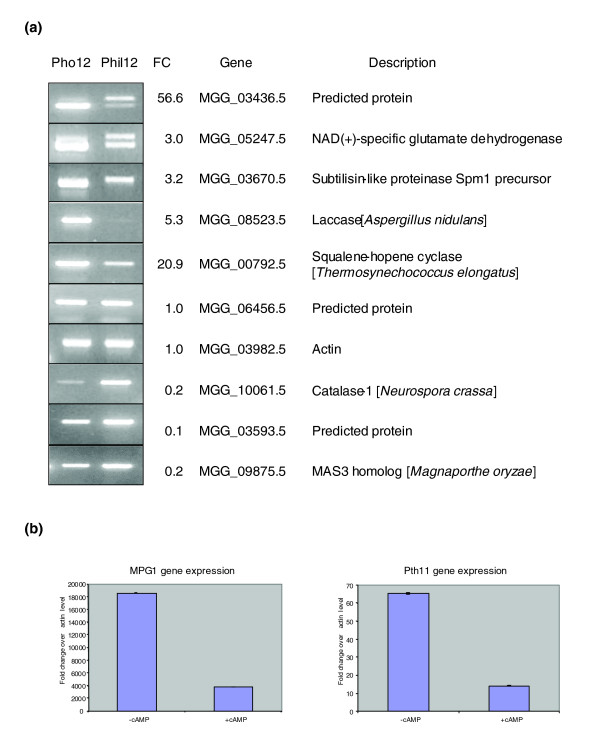
RT-PCR and qRT-PCR analysis of gene expression. **(a) **RT-PCR using RNA isolated from spores germinated on the hydrophobic (Pho12) and hydrophilic (Phil12) surfaces of GelBond after 12 h incubation compared to expression fold change (FC) derived from microarray data from the same time point. **(b) **qRT-PCR analysis of *MPG1 *and *PTH11 *using RNA from appressoria induced by cAMP (+cAMP) and germinating spores (-cAMP) after 9 h incubation on the hydrophilic surface of GelBond. Gene expression fold changes for *MPG1 *and *PTH11 *were 0.2 and 0.2, respectively, in our cAMP microarray study.

To confirm gene expression patterns derived from our microarray experiments, we performed reverse transcriptase PCR (RT-PCR) with five selected up-regulated genes, three down-regulated, and two showing no expression change (Figure 3). Genes were selected based on their overall expression levels, that is, represented high to medium to low expressed genes. If genes contain an intron, primers were designed to bridge the intron to distinguish amplification of transcript from any possible genomic DNA contamination (Additional data file 2). RT-PCR results were consistent with the microarray data, albeit the absolute levels of expression fold change showed slight variation (Figure 3). Two genes, *MPG1 *and *PTH11 *[44,45], were also subjected to analysis by quantitative RT-PCR (qRT-PCR). Both genes are required for pathogenesis. *MPG1 *has been shown to be highly expressed during appressorium formation [46]. However, our microarray and RT-PCR results indicated that both genes were more strongly up-regulated during germ tube elongation than appressorium formation (Figure 3).

### Appressorium consensus gene sets reveal key biological processes for appressorium formation

**Figure 4:**
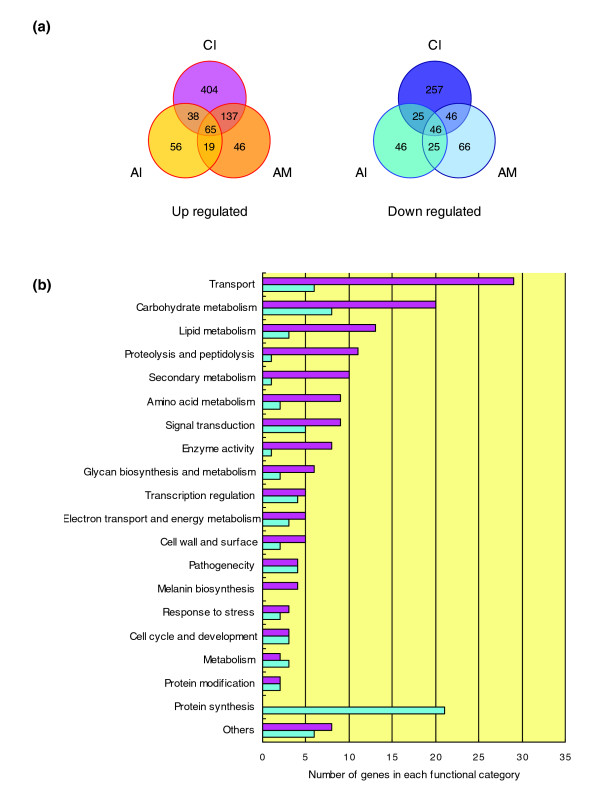
Functional categorization of appressorium consensus genes. **(a) **Appressorium associated expression profiles were combined and 240 up-regulated and 117 down-regulated genes were designated as appressorium consensus genes (in italics). Abbreviations are same as in Figure 2. **(b) **Up-regulated (in pink) and down-regulated (in blue) genes were grouped according to their putative function.

To identify genes that participate in appressorium formation, we compared gene expression profiles of appressorium initiation, maturation and cAMP induced appressoria. A total of 240 genes were up-regulated and 117 were down-regulated during appressorium initiation or maturation and in response to cAMP (Figure 4). These genes, referred to as appressorium consensus genes, were functionally grouped into GO categories based on manual curation as described in Materials and methods (Figure 4 and Additional data file 3). Overall, we noted a significant decrease in expression of genes involved in protein synthesis during appressorium induction. On the other hand, expression of genes associated with protein and amino acid degradation, lipid degradation, secondary metabolism, including melanin biosynthesis, and cellular transportation exhibited a dramatic upshift. Moreover, this set of genes exhibited nearly a four-fold enrichment for genes encoding secreted proteins. A detailed discussion of the functional groups exhibiting differential expression is presented below.

#### Major changes in amino acid and protein metabolism

Two of the major functional categories of genes in the up-regulated appressorium consensus gene set were those with predicted roles in protein and amino acid degradation. Protein sequence analysis of putative proteases recognized subgroups according to the active site and substrate specificity, such as acid proteases (MGG_03056.5, MGG_09032.5), aspartyl proteases (MGG_09351.5, MGG_00981.5), subtilisin-like proteases (MGG_03670.5, MGG_09246.5), calpain (calcium-dependent cytoplasmic cysteine proteinase)-like proteases (MGG_08526.5, MGG_03260.5), a cysteine protease required for autophagy (MGG_03580.5), a carboxylpeptidase (MGG_09716.5), and a tripeptidyl peptidase (MGG_07404.5).

**Figure 5:**
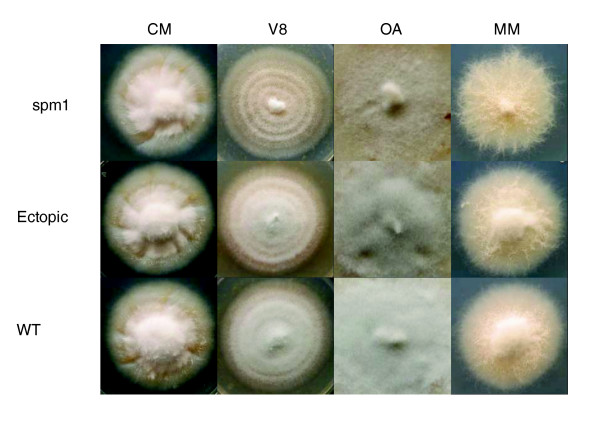
Vegetative growth of the *SPM1 *deletion mutant on various nutrient sources. *SPM1 *deletion mutant (Δspm1), ectopic strain and wild type 70-15 (WT) were incubated on solidified complete media (CM), oatmeal media (OA), V8 media (V8) and minimal media (MM) for seven days. Results shown are typical for all four independent *SPM1 *deletion mutants.

**Figure 6:**
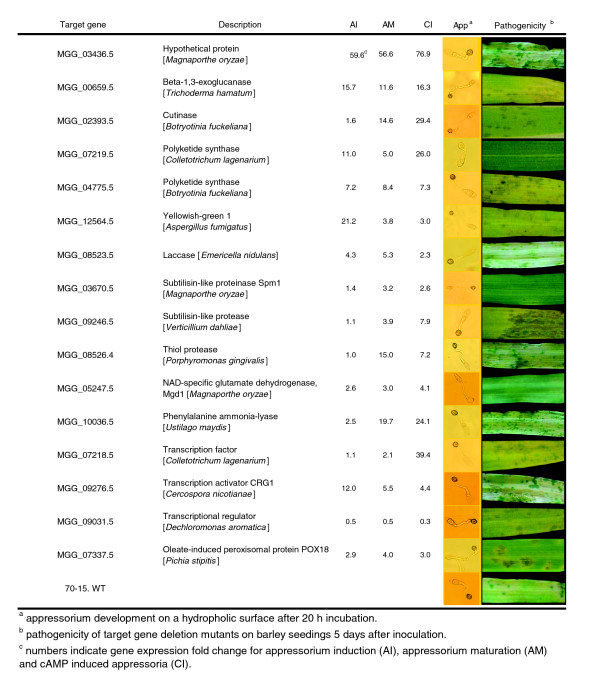
Appressorium formation and pathogenicity of targeted gene deletion mutants.

MGG_03670.5 (named *SPM1*) and MGG_09246.5 are putative proteases bearing the signature for subtilisin peptidase. A BLASTp search revealed that *SPM1 *and MGG_09246.5 have 39% amino acid identity and 55% similarity to each other and both match serine proteases from various microorganisms. The possibility of *SPM1 *as a pathogenicity candidate in *M. oryzae *was first proposed based on its prevalence in a cDNA library of mature appressoria [47]. *SPM1 *was also found to be abundant in SAGE tags derived from cAMP induced mature appressoria [39]. Although *SPM1 *contains a predicted signal peptide, the protein appears to be targeted to the vacuole [47]. As previously reported [48], *SPM1 *targeted deletion mutants produced melanized appressoria but exhibited severely reduced pathogenicity on rice and barley plants. Disease lesions failed to expand and sporulation was severely reduced [48]. In addition, further characterization here revealed vegetative growth, particularly aerial hyphae, of deletion mutants was decreased on the various nutrient sources such as oatmeal, V8 and minimal media but little difference was observed on complete media (Figure 5). On the other hand, targeted deletion mutants (see Materials and methods for details) of the putative protease encoded by MGG_09246.5 appeared normal and formed typical pigmented appressoria and developed disease symptoms on barley plants indistinguishable from wild type (Figure 6).

Protein degradation is highly regulated in many instances. Many short-lived proteins destined for degradation are selectively tagged by ubiquitin. It is noteworthy that several proteins involved in this process, including polyubiquitin (MGG_01282.5) and ubiquitin activating enzyme E1 like protein (MGG_07297.5), were up-regulated. Additionally, gene expression of putative ubiquitin protein ligases (MGG_11888.5, MGG_01115.5) exhibited increased expression in response to cAMP. Following selective tagging, proteins are degraded by the proteasome. Several probable 26S proteasome regulatory protein subunits (MGG_05477.5, MGG_05991.5, MGG_01581.5, MGG_07031.5) were up-regulated by cAMP. Currently, it is unknown which proteins are selectively tagged or how the proteasome regulatory proteins influence appressorium formation.

In addition to evidence for elevated protein degradation during appressorium formation, expression of genes involved in amino acid metabolism was also up-regulated. A putative aminotransferase (MGG_09919.5), a cystathionine γ-lyase (MG10380.5) that catalyzes the transition of cystathionine to cysteine, 2-oxobutanoate and ammonia, a cysteine dioxygenase (MG6095.5) in the cysteine degradation pathway, and a threonine deaminase (MGG_07224.5) required for threonine degradation were up-regulated. 2-Oxobutanoate (α-ketobutyrate), produced by cystathionine γ-lyase and threonine deaminase, can then be further metabolized through the tricarboxylic acid cycle. Turnover of the cellular storage amino acids, arginine and proline, to glutamate depends on the nutrient status of the cell. Genes involved in arginine and proline degradation to glutamate, such as arginase (MGG_10533.5), ornithine aminotransferase (MGG_06392.5), delta-1-pyrroline-5-carboxylate dehydrogenase (MGG_00189.5), and proline oxidase (MGG_04244.5), were up-regulated during appressorium formation.

NAD(+) dependent glutamate dehydrogenase (NAD-GDH) provides a major conduit for feeding carbon from amino acids back into the tricarboxylic acid cycle. The enzyme catalyzes the oxidative deamination of glutamate to produce α-ketoglutarate and ammonia (glutamate + NAD^+ ^→ α-ketoglutarate + NH_4 _+ NADH). Our gene expression data showed that the *M. oryzae *NAD-GDH homolog MGG_05247.5, which we have named *Mgd1*, was present in the up-regulated appressorium consensus gene set. Previous work using serial analysis of gene expression (SAGE) had shown that transcripts of *Mgd1 *were abundant in mature appressoria of *M. oryzae *induced by cAMP [39].

**Figure 7:**
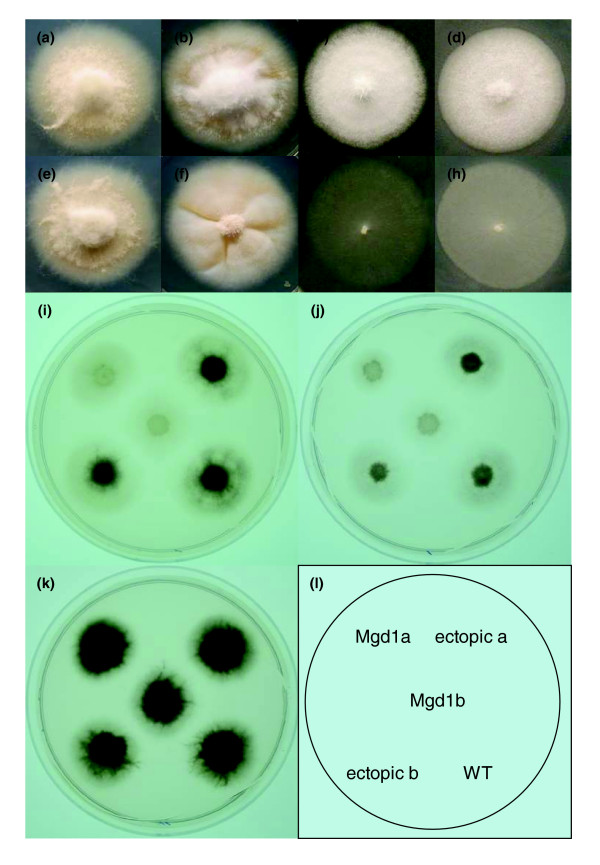
Vegetative growth of *Mgd1 *deletion mutants on various nutrient sources. **(a-h) **Wild type 70-15 (a-d) and *Mgd1 *deletion mutant (e-h) were grown on minimal media (a,e), complete media (b,f), minimal media with Tween 20 (c,g) or polyethylene glycol (d,h) as carbon source for seven days. Results shown are typical of all four independent *Mgd1 *deletion mutants. Results for ectopic strains were similar to wild type (data not shown). **(i-k) ***Mgd1 *deleted mutants (ΔMgd1a, ΔMgd1b), ectopic strains (ectopic a, ectopic b) and wild type 70-15 (WT) were grown for seven days on minimal media (0.125% glucose) with glutamine (i) and glutamic acid (j) as nitrogen source and minimal media (1% glucose) (k) as depicted in **(l)**. Photographs in (i-k) were taken on a light box to highlight differences in mycelial density.

To evaluate the function of *Mgd1 *in *M. oryzae*, we generated four independent targeted deletion mutants. Mutants lacked aerial hyphae when grown on complete media (Figure 7). In addition, growth was severely reduced on poor carbon sources such as Tween 20 and polyethylene glycol compared to ectopic and wild-type strains. The mutants also grow more poorly than ectopic and wild-type strains on glucose limiting conditions in the presence of glutamate and glutamine. NAD-GDH gene deletion mutants in yeast and *Aspergillus nidulans *also showed poor growth on glutamate as a sole nitrogen source [49,50]. To determine the role of *Mgd1 *in virulence, we evaluated mutants for appressorium formation and the ability to cause disease. Mutants had a reduced ability to form mature appressoria (45%) on an inductive surface; other appressoria appeared immature (41%) or were abnormal and highly swollen (4%) (Figure 6). When inoculated onto susceptible barley plants, the mutants exhibited highly reduced virulence and produced many fewer and smaller lesions (Figure 6). Thus, *Mgd1 *appears to be required for efficient metabolism of carbon and/or nitrogen from the break down of proteins under nutrient limiting conditions as experienced when cells are attempting to form appressoria.

In contrast to the activated expression of genes involved in protein and amino acid degradation, a major portion of the down-regulated genes encode components of the ribosome; 16 constitute the large ribosomal large subunit and 6 the small subunit (Figure 4 and Additional data file 3). Expression of all of these genes was up-regulated during spore germination and remained unchanged during germ tube elongation. However, upon appressorium induction the average level of expression fell 30% during appressorium initiation, and by more than 2-fold during appressorium maturation. Similar changes in gene expression patterns were observed in appressoria induction by cAMP.

#### Increased gene expression for lipid metabolism

Lipids are essential components of living cells as well as major sources of energy reserves. Several genes involved in the synthesis of the cell membrane components were induced during appressorium formation and they included a 7-dehydrocholesterol reductase (MGG_03765) that catalyzes the last step of cholesterol biosynthesis pathway, oxysterol binding protein (MGG_00853.5) involved in cholesterol biosynthesis, a probable sterol carrier protein (MGG_02409.5) involved in cholesterol trafficking and metabolism, glycerol-3-phosphate acyltransferase (MGG_11040.5) required for phospholipid biosynthesis, diacylglycerol cholinephosphotransferase (MGG_03690.5) involved in phosphatidylcholine biosynthesis pathway, and delta 8 sphingolipid desaturase (MGG_03567.5) involved in sphingolipid metabolism. Conversely, gene expression of fatty acid omega hydroxylases (MGG_10879.5, MGG_01925.5) and a cholinesterase (MGG_02610.5) involved in lipid degradation was found to be decreased.

Beta-oxidation of fatty acids in fungi occurs mainly in the peroxisome. Peroxisomes are membrane bound subcellular organelles where diverse anabolic and catabolic metabolisms, including peroxide metabolism, glyoxylate metabolism, and phospholipid biosynthesis, are conducted [51]. During fatty acid metabolism, the very long chain fatty acids (C22 or longer) are first transferred to coenzyme A by a very long chain fatty acyl-CoA synthetase. In *Saccharomyces cerevisiae*, very long chain fatty acyl-CoA synthetase, FAT1, disruption mutants showed reduced growth on media containing dextrose and oleic acid and very long chain fatty acids accumulated in cells [52]. Our data showed that a very long chain fatty acyl-CoA synthetase (MGG_08257.5) was up-regulated, suggesting fatty acid catabolism is involved in appressorium formation and function.

Recently, several genes for peroxisome structure, translocation of peroxisomal target proteins and metabolism in the peroxisome have been shown to be involved in pathogenicity, cellular differentiation and nutrient assimilation in fungi [53-56]. In *M. oryzae*, isocitrate lyase (*ICL1*) of the glyoxylate cycle, a *HEX1 *ortholog, *PTH2 *peroxisomal acetyl carnitine transferase, the multifunctional β-oxidation protein *MFP1 *and *MgPex6*, which is required for peroxisome biogenesis, were found to be necessary for functional appressorium development and fungal infection [30,57-59]. A putative fatty acid binding peroxisomal protein (MGG_07337.5) was identified in the up-regulated set of appressorium consensus genes. MGG_07337.5 encodes a protein with 40% identity and 59% similarity to the peroxisomal non-specific lipid transfer protein PXP-18, which is encoded by *POX18 *from *Candida tropicalis *and is highly conserved in filamentous fungi. *POX18 *mRNA was shown to be enriched by oleic acid and n-alkane rather than by glucose. PXP-18 appears to function in peroxisomal production of acetyl-coA either by guiding lipids through the oxidation processes or by protecting the acyl-coA oxidase enzyme [60-63]. To address the function of the PXP-18 homolog in *M. oryzae*, targeted deletion mutants were created. However, despite other evidence for the role of the peroxisome in appressorium formation and plant infection [30,53,64], the putative peroxisomal protein MGG_07337.5 was found to be dispensable for the development of a mature pigmented appressorium and disease symptom development on barley and rice plants in this study. Mutants were indistinguishable from wild type for other aspects of growth and development examined (Figure 6).

#### Carbohydrate metabolism: cell wall degradation, remodeling and carbon scavenging during appressorium development

Carbohydrates represent a major component of fungal biomass. Glycogen and various polyols are significant storage carbohydrates, whereas chitin, glucans and other polymers are primary constituents of the fungal cell wall. Inspection of our microarray gene expression analysis revealed a group of genes encoding enzymes for cell wall degradation, glucan mobilization and cell wall glycoprotein processing in the set of appressorium consensus genes. Several chitinase genes, such as MGG_00086.5 and MGG_01876.5, a beta-1,3 exoglucanase (MGG_00659.5), beta-glucosidase (MG10038.5), and polysaccharide dehydrogenase (MGG_01922.5), were up-regulated. However, other genes encoding glucan degrading enzymes showed opposite expression profiles. For example, glucan 1,4-alpha-glucosidase (MGG_01096.5), endo-1,4-beta-glucanase (MGG_05364.5), beta-1,3-glucosidase (MGG_10400.5) and alpha-L-fucosidase (MGG_00316.5) were down-regulated. The gene expression of a putative cell wall degrading protein (MGG_03307.5) containing a LysM associated with general peptidoglycan binding function and a glucosamine-6-phosphate deaminase (MG10038.5) in the glucosamine degradation pathway was increased but a UDP-N-acetylglucosamine-pyrophosphorylase (MGG_01320.5) that catalyzes the formation of UDP-N-acetyl-D-glucosamine, which is an essential precursor of cell wall peptidoglycan lipopolysaccharide, was repressed. These results suggest dynamic changes in glucan metabolism occur during appressorium formation.

Fungal cell walls contain high levels of mannoproteins (30-50% in yeast cell walls). These proteins are first glycosylated (glycosylphosphatidylinositol anchored) by mannosyltransferase adding mannose to their serine or threonine amino acid residues and then further processed by mannosidases. During appressorium development, several genes encoding these enzymes, alpha-1,2-mannosyltransferase (MGG_00695.5), beta-1,4-mannosyltransferase (MGG_10494.5), alpha-1,6 mannosyltransferase (MGG_03361.5) and alpha-mannosidase (MGG_00994.5) were up-regulated. In addition, gene expression of other homologs of an alpha-1,6-mannosyltransferase (MGG_00163.5), and a mannosidase (MGG_00084.5) were increased by cAMP treatment, implying that the active production of mannoproteins might aid to stabilize the cell wall during the rapid expansion of the appressorium. This hypothesis is supported by the finding that expression of two genes (MGG_03436.5 and MGG_02778.5), which encode putative mannosylated proteins, was strikingly increased. Expression of MGG_03436.5 was the most highly up-regulated in the appressorium consensus gene set (56.6-, 59.6-, 76.9-fold changes for appressorium initiation (AI), appressorium maturation (AM) and cAMP-induced appressoria (CI), respectively). MGG_03436.5 is a small hypothetical protein composed of 169 amino acids in 3 exons with no known functional domains. The deduced amino acid sequence showed 23% and 21% identity to that of *A. nidulans *cell wall mannoprotein MnpA (7.E-04) and *Aspergillus flavus *antigenic cell wall protein MP1 (1.E-04), respectively. Interestingly, a putative paralog of MGG_03436.5 in *M. oryzae*, MGG_02778.5, was also highly up-regulated in appressorium maturation and response to cAMP (15.5- and 15.1-fold changes for AM and CI, respectively). MGG_02778.5 was previously reported as the most abundant expressed sequence tag in a subtracted mature appressorium cDNA library [40]. The function of MGG_03436.5 was investigated by targeted mutagenesis. Surprisingly, no noticeable phenotypic differences, including appressorium formation and pathogenicity, in MGG_03436.5 gene deleted mutants were observed (Figure 6). This may suggest functional redundancy among related cell wall proteins. The *mnpA*-null mutant in *A. nidulans *showed an irregular outer cell wall layer; however, no phenotypic differences in spore germination, growth and cellular development were observed [65,66].

The expression of other genes involved in the utilization of non-preferred carbon sources was also up-regulated during appressorium formation. For example, coupled with increased expression of a transcriptional activator of alcohol metabolism (MGG_02129.5), an alcohol dehydrogenase I (MGG_03880.5) and a NAD^+^-aldehyde dehydrogenase (MGG_03263.5), which is involved in ethanol degradation for the production of acetyl-CoA, were up-regulated. Likewise, another gene involved in sugar alcohol degradation, L-arabinitol 4-dehydrogenase (MGG_01231.5), which hydrolyzes L-arabinitol to L-xylulose, was up-regulated. Gene expression of rhamnosidase A (MGG_05246.5), galactose oxidase (MG10878.5), a putative cytochrome P450 for alkane assimilation (MGG_05908.5) and lactate dehydrogenase (MGG_05735.5) was increased but expression of the dTDP-D-glucose 4,6-dehydratase in the rhamnose biosynthesis pathway (MGG_09238.5) and 2-isopropylmalate synthase in the leucine biosynthesis pathway (MGG_13485.5) were down-regulated.

#### Secondary metabolism during appressorium formation

Fungi produce an extensive array of secondary metabolites derived from a number of different biochemical pathways, including the polyketide, isoprenoid and shikimate acid pathways, as well as through modification of amino acids. Polyketides constitute a large class of secondary metabolites produced by filamentous fungi. They are synthesized from large multi-domain enzymes, polyketide synthases (PKSs) that share significant similarities to fatty acid synthases. Polyketide synthesis requires the coupling of malonyl-CoA to the elongating chain. It is noteworthy that the expression of MGG_07613.5, a putative acetyl-CoA carboxylase, the enzyme that catalyzes carboxylation of acetyl-CoA to produce malonyl-CoA, was up-regulated in the appressorium consensus gene set.

Melanin is one of the most thoroughly studied polyketides in *M. oryzae *and other pathogenic fungi. The three genes involved in the synthesis of dihydroxynaphthalene-melanin, a PKS, a synthalone dehydratase (SCD), and a hydroxynaphthalene reductase (THR), are clustered in the plant pathogenic fungus *Alternaria alternata *and the opportunistic human pathogen *Aspergillus fumigatus*. Targeted gene disruption experiments showed that these genes are essential for spore pigmentation and fungal pathogenicity [67-69]. In *M. oryzae*, the melanin biosynthesis genes *ALB1*, *RSY1 *and *BUF1 *are required for appressorium function but are not clustered [7,11]. *ALB1*, *RSY1 *and *BUF1 *correspond to MGG_07219.5 (a PKS), MGG_05059.5 (a SCD) and MGG_02252.5 (a THR), respectively. In our study,*ALB1 *and *RSY1 *genes were present in the set of appressorium consensus genes and were highly up-regulated (Additional data file 3). *BUF1 *was found to be up-regulated during appressorium initiation (3.9-fold change, *p *= 0.056) and significantly induced by cAMP (15.6-fold change, *p *< 0.001). All three genes were most highly induced during appressorium initiation, which is consistent with observations reported previously for their putative orthologs, *PKS1*, *SCD1 *and *THR1*, in the anthracnose fungus *Colletotrichum lagenarium *[70,71].

It is noteworthy that closer inspection of the genomic region on chromosome I containing the PKS *ALB1 *revealed the presence of a *BUF1 *homolog, MGG_07216.5. Positioned between these two genes is MGG_07218.5, a putative transcription factor. All three genes exhibited similar expression patterns during appressorium formation (Additional data file 4). MGG_07218.5 has an open reading frame of 1,926 nucleotides, potentially encoding a protein of 487 amino acids with a GAL4-like Zn2Cys6 binuclear cluster DNA-binding domain. A similar domain is also found in the *Pig1 *transcription factor (MGG_07215.5), previously reported to regulate mycelial melanin biosynthesis in *M. oryzae *[72]. *Pig1 *is located next to the *BUF1 *homolog (MGG_07216.5). However, *Pig1 *is not required for pathogenicity [72] and was not found to be differentially expressed in our experiments.

Although it is widely considered that the PKS gene MGG_07219.5 encodes ALB1, published evidence appears to be absent. Thus, to confirm that MGG_07219.5 corresponds to *ALB1*, targeted gene knock out mutants were created. Mutants exhibited the expected albino phenotype and were non-pathogenic on rice and barley. However, deletion of the putative transcription factor MGG_07218.5 resulted in no significant phenotypic changes in growth on various carbon and nitrogen sources or sporulation. In addition, mutants produced appressoria of normal appearance on a hydrophobic surface and produced disease symptoms on barley indistinguishable from wild type (Figure 6). Thus, this transcription factor does not appear to regulate melanin production, at least on its own. Examination of the promoter regions of *ALB1 *(MGG_07219.5) and the *BUF1 *homolog (MGG_07216.5) revealed putative GAL4 type transcription factor binding sites. GAL4 type transcription factors commonly form both homo- and heterodimers. Whether it is no more than a coincidence that these two transcription factors are physically associated with this genomic region or perhaps together both regulate melanin biosynthesis during appressorium formation awaits further investigation.

This genomic region on chromosome I also contains other genes associated with pigmentation. Next to the PKS *ALB1 *is a putative multicopper oxidase (MGG_07220.5), which is potentially orthologous to a spore pigmentation related gene in *A. fumigatus*, *abr1*. The signal intensity of MGG_07220.5 was low, but showed a significant increase during appressorium initiation. This region also includes a putative threonine deaminase (MGG_07224.5) and a putative peptide transporter (MGG_07228.5), both of which exhibited up-regulated expression in the appressorium consensus gene set. The melanin biosynthesis gene cluster in *A*. *fumigatus *also contains the genes for yellowish-green 1 (*ayg1*) and a laccase (*abr2*) in addition to *alb1 *(PKS), *arp1 *(SCD) and *arp2 *(THR) [69]. MGG_12564.5 is a hypothetical protein with 55% identity and 70% similarity to *ayg1 *and MGG_08523.5 has 36% identity and 55% similarity to *abr2*. Both MGG_12564.5 and MGG_08523.5 were significantly up-regulated during appressorium formation, although signal intensity was low. However, in contrast to reduced spore pigmentation in *ayg1 *and *abr2 *deletion mutants in *A. fumigatus*, targeted gene disruption mutants of MGG_12564.5 or MGG_08523.5 in *M. oryzae *resulted in no obvious phenotypic changes, including appressorium pigmentation and pathogenicity on barley plants compared to wild type (Figure 6).

In addition to the PKS *ALB1 *required for melanin biosynthesis, several other PKS genes involved in possible toxin biosynthesis were induced during appressorium formation. Increased gene expression was found for the putative PKS MGG_04775.5, which appears to be the ortholog of PKS1 and PKS2 required for T-toxin production in the maize leaf blight fungus *Cochliobolus heterostrophus*. The genomic neighborhood around MGG_04775.5 contains a serine hydrolase (MGG_04774.5), an ABC transporter (MGG_13762.5), and a polyphenol oxidase (MGG_13764.5). These clustered genes were all up-regulated in the appressorium consensus gene set except MGG_13762.5, which was only up-regulated on the hydrophobic surface. Similar to the Tox1 locus in *C. heterostrophus *[73], which contains two PKS genes, MGG_04775.5 was found to be closely located with another PKS1 homolog, MGG_13767.5. However, this gene exhibited no significant changes in gene expression. Expression of MGG_07803.5, another PKS gene, also did not change significantly; however, several neighboring genes, MGG_07784.5, MGG_07785.5, a manganese peroxidase (MGG_07790.5), an oxidoreductase (MGG_07793.5) and a major facilitator superfamily transporter (MGG_07808.5) were induced during appressorium formation. It is possible this cluster of genes directs the synthesis of an unknown secondary metabolite.

Transcript levels of MGG_10072.5, which has 69% identity with PKSN required for alternapyrone biosynthesis in the early blight fungal pathogen *Alternaria solani*, was dramatically increased during appressorium maturation and cAMP treatment (52- and 100-fold change, respectively). Interestingly, the genomic region containing MGG_10072.5 also contains a putative FAD-dependent oxygenase (MGG_13597.5) and two cytochrome P450s (MGG_10070.5, MGG_10071.5), which is very similar to the genomic organization containing the PKSN locus in *A. solani *[74].

HMG-CoA synthase (3-hydroxy-3-methylglutaryl-coenzyme A synthase) and HMG-CoA reductase (3-hydroxy-3-methylglutaryl-coenzyme A reductase) catalyze the conversion of acetoacetyl-CoA to HMG-CoA and HMG-CoA to mevalonate, the limiting steps for the synthesis of isoprenoids, such as cholesterol and egosterol as well as numerous secondary metabolites, via the mevalonate pathway. Both HMG-CoA synthase (MGG_01026.5) and HMG-CoA reductase (MGG_08975.5) were up-regulated in the appressorium consensus gene set. Intriguingly, a PKS (MGG_08969.5), a regulatory enzyme (MGG_08974.5) and a secondary metabolite transporter (MGG_08970.5) flank MGG_08975.5. The expression levels of these genes were not significantly changed. However, in other fungi that contain similar arrangements of apparently orthologous genes, these genes confer important regulation and biological properties. In *Penicillium citrinum*, the orthologous genomic region contains a cluster of genes that synthesize ML-236B (compactin), a lovestatin-like inhibitor of HMG-CoA reductase [75]. Furthermore, MGG_08969.5 appears orthologus to *NPS6*, a gene required for fungal virulence and resistance against oxidative stress in plant pathogenic ascomycetes fungi [76,77], suggesting that this gene cluster may play an important role in the pathogenicity of *M. oryzae*.

Several other genes involved in secondary metabolism were found in the up-regulated appressorium consensus gene set. For example, MGG_00385.5 and MGG_00573.5 encode proteins homologous to an ochratoxin-A non-ribosomal peptide synthetase in *Penicillium tetracenomycin *and an O-methyltransferase involved in polyketide synthesis in *Streptomyces glaucescens*, respectively. Other examples include MGG_06585.5 and MGG_04911.5, which are respectively similar to a putative short-chain dehydrogenase/reductase, Fum13p, and a putative cytochrome P450 monooxygenase, Fum15p, in the fumonisin biosynthesis gene cluster of the maize ear rot fungus *Gibberella moniliformis*. Genes encoding other key enzymes catalyzing critical steps in secondary metabolism were also up-regulated. For example, phenylalanine ammonia lyase (PAL; MGG_10036.5), which catalyzes the non-oxidatative deamination of phenylalanine to cinnamic acid and ammonia, the opening reaction in the phenylpropanoid pathway that generates various precursors of secondary metabolites such as toxins, antibiotics and pigments, was in the set of up-regulated appressorium consensus genes. Other important genes for secondary metabolism such as squalene hopene cyclase (MGG_00792.5), a key enzyme for hopanoid (triterpenoid) biosynthesis and isoflavone reductase (MGG_06539.5), a key enzyme in phenylpropanoid biosynthesis, were up-regulated.

The identification of several genes central to secondary metabolism with elevated expression strongly implies the existence of appressorium specific fungal metabolites that may play a role in establishing the pathogenic interaction. For example, it has been shown that the host selective toxin (HC-toxin) synthesis is highly induced during appressorium development in the maize leaf spot pathogen *Cochliobolus carbonum *[78,79]. To investigate the role of secondary metabolites in appressorium formation and function, we selected two key genes for functional analysis, the PKS ortholog of PKS1 (MGG_04775.5) required for T-toxin in *C. heterostrophus *and the PAL gene (MGG_10036.5). However, unlike PKS1 and PKS2, which are required for T-toxin production and high virulence of *C. heterostrophus *[73], targeted knock out mutants of MGG_04775.5 were indistinguishable from the wild type, were able to form appressoria and were pathogenic towards barley and rice. Likewise, deletion mutants of MGG_10036.5 appeared to have a normal phenotype in growth, development and pathogenicity compared with the wild type (Figure 6). PAL was also found to be dispensable for sexual development and virulence in the maize smut fungal pathogen *Ustilago maydis *[80].

#### Transporters

Fungal transporters play an essential role in pathogenicity by exporting host-specific and non-host-specific secondary metabolites, including toxins, into the host plant tissue or provide a protective role by removing plant defense compounds or disease control agents from the fungal cell [81]. During appressorium development, the expression of 35 transporters with various substrate specificities were differentially up-regulated with the most striking being transporters related to toxin export and ion transport.

During appressorium induction, gene expression of 11 transporters was increased whereas in mature appressoria, 25 transporters were up-regulated. Up-regulated transporters included several from the major facilitator superfamily, such as MGG_02167.5, MGG_01778.5, MGG_10869.5, MGG_06794.5, MGG_03640.5 and MGG_03843.5, which share close homology with toxin efflux pumps and multidrug transporters as well as MGG_07062.5 and MGG_09827.5, and MGG_04511.5 and MGG_00275.5, which appear to be involved in exporting monocarboxylate and nicotinamide mononucleotide, respectively. Other up-regulated transporters included the ABC transporter (MG10410.5), which is homologus to mdrA2 in *Dictyostelium discoideum*, and MGG_06604.5, a homolog of human CLN3, which is involved in the ATP-dependent transport of arginine into the vacuole, and putative peptide trasporters MG10200.5, MGG_07228.5 and MGG_08258.5. In contrast, gene expression of several putative carbohydrate transporters (MGG_04927.5, MGG_09193.5, MGG_03298.5, MGG_07843.5, and MGG_08968.5) was down-regulated, as might be expected due to the lack of nutrients in the surrounding environment.

Enhanced gene expression was also detected in a group of putative ion transporters, such as a K^+ ^transporter (MGG_02124.5), a cation efflux pump (MGG_07494.5), a CorA-like Mg^2+ ^transporter (MGG_02763.5), P-type ATPases (MGG_00930.6 and MGG_04852.5) and other ion transporters (MGG_05085.5 and MGG_04105.5). A putative large conductance mechanosensitive channel (MGG_01489.5) was down-regulated during appressorium formation.

Several fungal transporters have been recognized to play a role in cellular development and are regarded as virulence factors. In *M. oryzae*, an ATP driven efflux pump, *ABC1 *(MGG_13624.5) was strongly induced by azole fungicides and the rice phytoalexin sakuranetin. Mutants lacking *ABC1 *were unable to colonize host tissue [82]. Likewise, deletion of the multidrug resistance transporter *ABC3 *(MGG_13762.5) led to complete loss of pathogenicity, although appressorium formation was unaffected [36]. Deletion mutants of *Pde1*, a P-type ATPase, were impaired in appressorium development and pathogenicity [83]. In our experiments, no significant changes in gene expression of *ABC1 *and *Pde1 *were detected during appressorium development. However, MGG_04852.5, the closest homolog of Pde1 (50% identity) was present in the up-regulated appressorium consensus set. *ABC3 *was highly induced during early stages of appressorium development on the hydrophobic surface.

#### Elevated vesicle transport and secreted proteins

The vesicular secretory pathways have not been well studied in plant pathogenic fungi; however, increased expression of genes involved in membrane trafficking and signal transduction was apparent during appressorium formation. Up-regulated genes included a homolog of yeast phosphatidylinositol transfer protein, Sec14p, for vesicle budding from the Golgi complex (MGG_00871.5), a putative phospholiphase-D for coated vesicle formation (MGG_05804.5), a putative dynamin GTPase for vesicle detachment from membrane (MGG_08732.5), and putative lipid binding proteins with a C2 domain, a Ca^2+^-dependent membrane-targeting module for signal transduction or membrane trafficking (MGG_09947.5 and MGG_01150.5). Another putative transmembrane protein with a C2 domain, MGG_01094.5, was down-regulated. The up-regulation of a number of genes associated with vesicle transport and secretion is consistent with our observation that expression of secreted proteins was enriched during appressorium formation.

Secreted proteins are likely to be key determinants of host fungal pathogen interactions [84-88]. The overall percentage of putative secreted proteins in the *M. oryzae *proteome is 7%. However, about 26% of appressorium consensus genes contain proteins with translocation signals and include several previously characterized pathogenicity-related genes such as *GAS *(gEgh16 homologs expressed in appressorium stage) homologs and hydrophobin proteins. *GAS3 *(MGG_04202.5) and *GAS1 *(MGG_12337.5) were previously shown by differential hybridization analysis to be highly abundant in an appressorium-specific cDNA library [89]. Deletion mutants developed appressoria but showed reduced pathogenicity on rice and barley leaves [89]. We found that both *GAS1 *and *GAS3 *were highly up-regulated throughout appressorium formation. In addition, other *GAS *homologs (MGG_02253.5 and MGG_09875.5) were also differentially expressed. During appressorium morphogenesis, MGG_09875.5, the closest paralog to *GAS1 *(62% identity), was strongly down-regulated (0.1-, 0.2-, 0.2-fold change in AI, AM and CI, respectively) while MGG_02253.5, the closest paralog to *GAS3 *(45% identity) was down-regulated during AI but up-regulated in mature and cAMP induced appressoria (0.3-, 2.5-, 2.7- in AI, AM and CI, respectively).

The hydrophobin *MPG1 *(MGG_10315.5) was previously shown to be highly expressed in infected leaves compared to growth in complete media. Further, deletion mutants produced less appressoria and exhibited reduced pathogenicity [45]. In this study, similar to *PTH11*, gene expression of *MPG1 *was strongly down-regulated on the hydrophobic surface and by exogenous cAMP treatment compared to germ tubes that continued to develop vegetatively. We also observed that the expression profile of another extracelluar protein SnodProt1 homolog (MGG_05344.5), which has some physical properties in common with hydrophobins, was also down-regulated during appressorium formation. SnodProt1 is a member of the cerato-platanin family, which in *Phaeosphaeria nodorum *was shown to be involved in pathogenicity and plant defense response in wheat [90]. Recently, the SnodProt1 homolog in *M. oryzae *has been functionally characterized [91]. Targeted deletion mutants exhibited reduced pathogenicity and impaired *in planta *growth, but there was no evidence that purified SnodProt1 protein had phytotoxic properties as suggested for other fungal homologs [92].

Other secreted proteins have hydrolytic enzyme activity potentially involved in degradation of the host cell wall and may play some role in appressorium development and function. For example, putative exogenous cutinases (MGG_02393.5, MGG_11966.5) and a fungal lignin peroxidase (MGG_07790.5) were highly up-regulated. Until recently the role of cutinase was largely discounted based on the finding that the cutinase *CUT1 *(MGG_01943.5) is not essential for pathogenicity in *M*. *oryzae *[93]. However, expression of this gene was not up-regulated in our experiments. Skamnioti and Gurr [94] reported that *CUT2 *(MGG_09100.5) is required for full virulence. As reported by Skamnioti and Gurr [94], we also found this gene was highly up-regulated during late stages of appressorium formation on the hydrophobic surface. To further evaluate the role of cutinases, we investigated the function of the putative cutinase MGG_02393.5 because this gene was also up-regulated by both hydrophobic and cAMP. However, gene deletion mutants exhibited no observable changes in virulence or other phenotypic differences (Figure 6).

#### Cell signaling pathways

In addition to cAMP, several cell signaling pathways have been shown to be involved in regulating appressorium formation. Calcineurin is a Ca^2+^/calmodulin-dependent serine/threonine phosphatase that is involved in many signal pathways for cation homeostasis, cell differentiation, morphogenesis, cell wall integrity and pathogenicity [95-98]. Phosphorylation activity of calcineurin is inactivated when it is coupled with cyclophilin and other FK506-binding proteins (FKBPs) in the presence of inhibitors such as cyclosporine A and tacrolimus (FK506). In *M. oryzae*, cyclophilin (*CYP1*; MGG_10447.5) has been shown to be involved in fungal virulence and appressorium turgor generation [99]. Cyclosporine A inhibits hyphal growth and appressorium formation in a *CYP1*-dependent manner, supporting a role for calcineurin in regulating appressorium development. The transcriptional activity of calcineurin (MGG_07456.5) remained unchanged; however, expression of MGG_06035.5, a putative ortholog of FKBP12, decreased. In addition, MGG_01150.5, a putative ortholog of CTS1 (calcineurin temperature suppressor), which is required for the full virulence of *Crytococcus neoformans*, was up-regulated during appressorium formation.

Many external signals are transmitted to the inner workings of the cell through receptors that are embedded in the plasma membrane. During appressorium induction, several genes encoding putative G-protein coupled receptor-like proteins with seven membrane spanning domains (PTH11 MGG_05871.5, MGG_02692.5, MGG_05214.5, MGG_10571.5) were significantly down-regulated, although the expression of a CFEM-containing protein (MGG_09570.5; CFEM is an eight cysteine-containing domain present in fungal extracellular membrane proteins) was up-regulated. Also, expression of another CFEM protein, ACII (MGG_05531.5), which interacts with MAC1 adenylate cyclase in cAMP signaling pathways, was very intensive and was up-regulated in young appressoria but was dramatically reduced in mature appressoria and was significantly down-regulated by cAMP. We also observed increased gene expression of MGG_00438.5, which encodes a putative transmembrane protein, PAT 531. Previous studies reported that deletion of this gene resulted in reduced pathogenicity of *M. oryzae *towards weeping lovegrass [100].

## Discussion

In this study, we performed microarray studies to identify genome wide fluctuations in gene expression during germination, appressorium induction and maturation. We then used these data as a guide to identify and subsequently characterize core biological processes, in some cases previously unrecognized processes required for infection related development and pathogenesis. Key to this study was the experimental design. First, we performed a direct comparison of gene expression during spore germination on an inductive surface verses a non-inductive surface. Second, we compared the expression patterns with those obtained from a direct comparison of conidia germinating in the presence of cAMP verses its absence. The integration of these data sets revealed a core set of appressorium-induced genes common to the different stimuli. Subsequent bioinformatics and functional analyses of these so-called consensus genes shed new light on the biochemical processes required for appressorium formation and function.

Prior to this study, no genome-wide transcriptional profiles related to infection-related morphogenesis have been published for filamentous fungi. A few studies using partial gene sets have provided some insights into fungal pathogenesis [41,42,101,102]. However, due to a number of reasons, including experimental design, scale of experiments, analytic procedures applied, as well as lack of supporting functional evaluation, drawing generalized conclusions must be approached cautiously. Nevertheless, our data reveal a number of key features and represents an important benchmark for other future studies of pre-infection-related development. Very little had been known regarding the role of protein degradation and amino acid metabolism in appressorium formation and function. We found that during appressorium formation a number of genes required for protein metabolism were differentially expressed at significant levels. In particular, several proteases, including the putative vacuolar subtilisin-like protease *SPM1*, which is required for penetration and *in planta *growth, were up-regulated specifically during appressorium formation. Our data also revealed that genes involved in targeting proteins for degradation as well as the machinery involved in protein degradation were also up-regulated. Several proteases contained an export signal, suggesting they may be secreted and act as virulence factors as has been shown for homologs in related fungi [103]. Coupled with elevated expression of genes for protein degradation, we found genes involved in amino acid metabolism to be stimulated during appressorium formation. For example, a NAD-dependent glutamate dehydrogenase (*Mgd1*) was significantly up-regulated in a manner very similar to *SPM1*. *Mgd1 *deletion mutants were unable to make normal appressoria and showed significantly reduced virulence. Microarray analysis of 1,730 genes from the insect fungal pathogen *Metarhizium anisopliae *also revealed up-regulation of a number genes involved in protein metabolism, including several proteases, as well as amino acid catabolism during appressorium formation on insect cuticle [102,104,105]. It should be pointed out that proteins represent a major component of insect cuticle and, thus, induction of proteases was not unexpected. Several proteases, including subtilisins, were also up-regulated during starvation conditions, but less so than on insect cuticle [104]. Others were specifically up-regulated on cuticle. The authors speculate that proteases may 'sample' the cuticle, resulting in further induction for nutrition and possibly to aid in entering the host. This, however, has not been formally demonstrated. Also, similar to our observations in *M. oryzae*, an NAD-GDH was up-regulated in *M. anisopliae *on insect cuticle, possibly in response to elevated levels of amino acids released through protein hydrolysis and uptake. In yeast, NAD-GDH is repressed by ammonium and derepressed by glutamate and carbon starvation [49,106,107]. However, iHOncreased enzyme activity was observed during yeast to mycelium morphogenesis in *Mucor racemousus *and *Benjamineilla poitrasii *and hyphal adhesion and arial hyphae development in *Neurospora crassa *[108-110], suggesting NAD-GDH plays a critical role in development. In sum, for *M. oryzae*, the combination of global gene expression analysis and subsequent functional interrogation revealed a direct connection between protein catabolism, carbon and nitrogen scavenging from amino acids and appressorium formation and function.

In recent years, attention has focused on effector molecules, many of which are secreted proteins or secondary metabolites. These molecules may serve as virulence determinants or help shield the fungus from being detected by its host. In some cases, the host may recognize these molecules and activate its defense mechanisms. For example, the *M. oryzae *avirulence gene *ACE1 *encodes a PKS [85,111]. In this case, it is presumed that the metabolite synthesized by this gene product confers biological function. Other avirulence genes such as *avr-pita *encode a small-secreted protein that has been shown to directly interact with its cognate resistance gene product, Pita, in rice [112]. Secreted effector proteins identified in oomycetes share the common motif, RXLR, which is essential for their translocation into host plant cells [88]. Enriched in the apoplast of tomato leaves, the chitin binding protein Avr4 from *Cladosporium fulvum *protects the fungal cell wall from the attack by plant chitinases [87]. During colonization of tomato xylem vessels, the root invading fungus *Fusarium oxysporum *secretes a number of small cysteine-rich proteins, such as SIX1, which are recognized by host resistance gene products [86]. Whole genome expression studies using the cereal head blight fungus *Fusarium graminearum *Affymetrix GeneChip revealed numerous genes encoding potential plant cell wall degrading enzymes, including xylanases, mannanases, pectinases, glucanases, galactosidases and cutinases, as well as genes involved in trichothecene biosynthesis, were expressed *in planta *[113]. Thus, it is reasonable to expect that during pre-penetration phases of the host-pathogen interaction, *M. oryzae *would begin to mobilize effector molecules. Indeed, during appressorium formation, we detected a nearly four-fold enrichment of genes encoding products with a putative signal peptide in the up-regulated consensus gene set as well as increased expression of numerous genes associated with secondary metabolism and secretion. GAS1 and GAS3 were among the most highly expressed secreted proteins. Stage-dependent gene expression of GAS homologs was previously reported in the cereal powdery mildew fungus *Blumeria graminis*. *Egh16H1 *was strongly induced during haustorium formation whereas *gEgh16 *appears to be more induced during spore germination [114]. The function of these genes in *B. graminis*, however, has not been determined. A number of secreted proteins, predominantly hydrolases such as proteases and chitinases, were up-regulated during appressorium formation by *M. anisopliae *on insect cuticle [104]. Although these enzymes are likely involved in nutrition, it is possible they release effector molecules. Chitinase and parts of the secretion apparatus were also found to be up-regulated in *B. graminis *during the pre-penetration stages compared to conidial germination, which presumably would promote delivery of any effector molecules [101]. Indeed, a number of genes encoding small secreted metallothioneins were found to be strongly expressed during haustoria formation by the rust fungus *Uromyces fabae *compared to germinated uredospores [115]. We also found several examples of co-expressed genes clustered around PKS genes, suggesting they may synthesize pathogenesis-associated metabolites. Several phytotoxic secondary metabolites have been isolated from *M. oryzae*, such as pyricularin and picolinic acid, but little is known regarding the genes that are required for their synthesis [116]. This is a research topic that would appear to warrant further investigation.

Somewhat surprisingly, some genes known to be essential for appressorium development appeared to be down-regulated in our expression studies. The importance of MPG1 (MGG_010315.5), a hydrophobin, and PTH11 (MGG_05871.5), a membrane spanning protein in appressorium development, are well documented, but our data showed they were more highly expressed in spores germinating on a hydrophilic surface than in incipient appressoria. This may suggest that the gene products are required initially and perhaps transiently for subsequent morphological changes and is consistent with a role in surface sensing and attachment. Once the environmental cue is detected and the intracellular signal pathways activated, these proteins may no longer be needed for appressorium formation to proceed.

In addition, our data also revealed that a large set of ribosomal protein genes were down-regulated during appressorium formation. Similar observations were reported for *M*. *anisopliae *during appressorium formation on insect cuticle and in the animal pathogens *C*. *neoformans *and *Candida albicans *early in pathogenesis following internalization by macrophages [104,117,118]. This pattern of down-regulation of genes associated with ribosome biogenesis has been observed commonly in a number of organisms subjected to nutrient starvation or upon treatment with rapamycin, a potent inhibitor of target of rapamycin (TOR) kinase [119]. In *M. oryzae*, this set of genes, with the exception of MGG_05716.5, was down-regulated significantly (>2-fold) when cells were shifted to media lacking nitrogen for 12 hours [48]. In the experiments reported here, spores were germinated in water and thus would be starved for both nitrogen and carbon; however, it was only upon appressorium formation that expression of genes associated with ribosome biogenesis fell. The role of TOR is complex, it is not only associated with starvation but with development. In yeast, TOR interacts with a number of other proteins, including STE20, and regulates sexual development [120]. In addition, rapamycin is a potent inhibitor of filamentous growth in fungi, including *A. fumigatus *[121]. Thus, during appressorium formation, the TOR pathway may be involved in redirecting resources away from polar growth to breaking down cellular components in order to generate materials necessary for the penetration process to be effective.

While perturbation of any one signal cascade is often sufficient to affect appressorium formation, considerable evidence suggests that there is cross-talk between the signal pathways. Previous studies have shown that expression of *GAS1 *and *GAS2*, which are highly expressed during appressorium formation, requires *Pmk1*, suggesting they are regulated by the MAPK pathway [89]. The presence of these genes in the appressorium consensus gene set is consistent with cross-talk between the cAMP and MAPK pathways. In addition, our microarray data suggest the involvement of the calcineurin dependent and TOR signal pathways in regulating appressorium formation. Clarifying the role and interconnection of these different signal pathways may be served by examining gene expression in different mutant backgrounds, particularly within the first few hours of germination.

While gene expression analysis is a useful tool to identify genes associated with a particular process, it is by no means definitive. In a recent report, 250 *M*. *oryzae *mutants defective in appressorium formation and/or pathogenesis were created by agrobacterium-mediated random insertion mutagenesis [122]. The majority of the mutation sites were located in intergenic regions. Interestingly, most of the genes flanking the insertion sites showed no differential expression changes in our microarray experiments. Moreover, of 90 genes with well-characterized roles in pathogenicity in *M. oryzae*, the vast majority (81) were not up-regulated during appressorium formation. For example, lipid metabolism has been strongly linked to generating the high levels of glycerol found in appressoria and triacylglycerol lipase activity has been reported to be induced during appressorium formation [9]. However, in our microarray study, only one (MGG_00528.5) of seven genes encoding this activity showed any evidence of induction and even then expression levels were low. In addition, we observed that *MFP1*, the gene that encodes a multifunctional beta-oxidation protein involved in lipid degradation, was more highly expressed in spores than during germination or appressorium formation. Also, homologs of genes involved in peroxisome biosynthesis, such as *MgPex6*, exhibited little indication of being up-regulated during appressorium formation. Similarly, we found limited evidence for genes involved in autophagy being up-regulated during appressorium formation. Both *MgATG1 *or *MgATG8 *were induced slightly during germination, but showed little or no further induction during appressorium formation.

On the other hand, the majority of mutants generated in our study, which were genes highly up-regulated during appressorium formation, retained their ability to differentiate infection structures and cause disease. It is possible that the lack of an observable phenotype variant in many of our gene knock-out mutants may be due to functional redundancy among possible (unidentified) paralogs or the activation of alternative compensatory pathways. For example, *M. oryzae *appears capable of compensating for the loss of MGG_02393.5, but appears to be unable to fully compensate for the loss of *CUT2*, even though both homologs exhibited similar expression patterns. Deleting *SPM1 *had a dramatic effect on sporulation and virulence, but deleting its paralog, MGG_09246.5, showed no detectable phenotypic changes despite both genes having very similar expression patterns. Thus, *M. oryzae *is able to fully compensate for the loss of some gene homologs but not others. This suggests some level of functional specificity may exist. Functional redundancy/compensation appears to be commonly encountered in biological processes. For example, only about 10% of genes down-regulated by genome-wide RNA interference provided phenotypic changes in *Caenorhabditis elegans *[123]. In *S. cerevisiae*, less than 7% of genes that exhibited a significant increase in gene expression were found to be required for optimal growth under the tested conditions. Moreover, many genes that were necessary for the fitness were not differentially expressed. [124]. To address issues related to functional redundancy in *M. oryzae*, employing RNA interference to silence all members of a gene family may be effective. In other situations, it may be valuable to explore the use of over-expression to evaluate the role of particular genes.

## Conclusion

Genome-wide expression profiling during spore germination and appressorium formation revealed that the blast pathogen *M. oryzae *undergoes significant changes in gene expression during infection-related cellular differentiation. Functional analysis and characterization of the differentially expressed genes provides new insight into appressorium morphogenesis, in particular regarding the role of protein degradation for appressorium function. We provide a comprehensive list of genes that might be involved in appressorium formation and function solely or in combination with other genes. Our data will be beneficial for further studies on fungal pathogenesis, including gene expression studies in other fungal pathogens. We expect the emergence of additional functional information regarding *M. oryzae *genes with currently no known biological function will help broaden the scope of future analyses.

## Materials and methods

### Appressorium induction by physical cue and exogenous cyclic AMP

*M. oryzae *strain 70-15 spores were collected from ten-day-old V8 agar plates and adjusted to 10^5 ^conidia/ml. Spore suspensions were placed on the appressorium-inducing (hydrophobic) and non-inducing (hydrophilic) surface of GelBond (Cambrex Bio Science Inc., Rockland, ME, USA) at 6.25 × 10^4 ^spores/cm^2 ^and incubated at 25°C in the dark and for 7 and 12 h. By 7 h, on the hydrophobic side of the GelBond film, essentially all spores germinated and tips of germ tubes had started to hook and swell, forming young appressoria. These young light-colored appressoria developed into dark mature appressoria at 12 h. On the hydrophilic side of the GelBond, germ tubes continuously elongated and branched several times through 12 h with no other developmental changes. Appressoria were also induced by adding exogenous cAMP (final concentration of 50 mM) to the spore suspension placed on the hydrophilic surface of GelBond as described above. In the presence of cAMP, melanized appressoria were evident at 9 h (Figure 1a).

### RNA sample preparation and microarray data collection

RNA was purified following standard protocols. Briefly, following incubation, fungal material was flash frozen with liquid nitrogen, scrapped from the support, and ground in liquid nitrogen. Total RNA was extracted using Qiagen RNAeasy Mini kit (Qiagen Inc., Valencia, CA, USA) according to the manufacturer's protocol. RNA was also extracted from spores in water immediately after being collected from agar plates. Quality analysis and quantification were performed using the Agilent Bioanalyzer (Agilent Technologies, Inc., Wilmington, DE, USA) and the Nano Drop (NanoDrop Technologies, Inc., Wilmington, DE, USA).

RNA from the two biological replicates was pooled in equal amounts and 1 μg was labeled by reverse transcriptional incorporation of cyanine 3 (Cy3) or cyanine 5 (Cy5) labeled dCTP, using an oligo(dT) primer. The Agilent Fluorescent Linear Amplification Kit protocol for fluorescent cRNA synthesis was used. The concentration and the dye incorporation of labeled cRNA were measured by Nano Drop. According to the microarray hybridization scheme (Figure 1b), equimolar amounts of cRNA samples, each labeled with Cy3 or Cy5, were co-hybridized to the *M. oryzae *Oligo Microarray (Agilent technology, #G4137A) using the Agilent 60-mer-oligo microarray processing protocol as previously described [48]. Following drying, slides were immediately scanned with an Agilent G256BA microarray scanner. Image files from the scanner were analyzed with the Agilent 2567AA Feature Extraction software (version 1.6.1.1). For signal normalization, the output from Agilent Feature Extraction was first converted into GPR format to conform to the input format requirements for the 'Bioconductor/Aroma' software [125]. The data were read into Aroma and 'within slides LOWESS normalization' and 'across slides LOWESS normalization' methods were applied. After normalization, the data were extracted for each treatment - each having four replicates, two from Cy3 and two from Cy5, from which the mean and other statistics were calculated.

### Gene expression profiles and correlation analysis

Gene expression profiles for appressorium initiation, appressorium maturation and appressorium induced by cAMP were determined by comparing the hybridization signals of RNA harvested from the hydrophobic surface after 7 and 12 h incubation (Pho7 and Pho12, respectively) and the hydrophilic surface after 9 h incubation with cAMP (cAMP9), directly to those from the hydrophilic surface after 7 and 12 h incubation (Phil7 and Phil12, respectively), and the hydrophilic surface after 9 h incubation without cAMP (Phil9). The expression profile during germ tube elongation was made by comparing the hybridization signals of RNA harvested from the hydrophilic surface after 12 h (Phil12) to those from the hydrophilic surface after 7 h (Pho7). To construct the gene expression profile for spore germination, the hybridization signals of RNA harvested from the hydrophilic surface after 7 h incubation (Phil7) were compared to those of RNA from ungerminated spores (Spore). For each profile, the ratio of the expression level for the 10,176 *M. oryzae *probes was calculated. The log_2 _-transformed value of the ratio was used for pairwise correlation analysis between expression profiles. Genes were designated as differentially expressed if their average signal intensities were equal or above 200 in at least one condition and their expression ratios were equal or greater than 2-fold with *p *< 0.05 (Student's *t*-test).

### RT-PCR and quantitative RT-PCR

RT-PCR as described previously was conducted on selected genes that were either significantly up-regulated, down-regulated, or showed no difference in their expression profile in the microarray experiments [48]. PCR was performed on independently generated templates using primers shown in Additional data file 2. Total RNA was prepared from germinating spores on hydrophilic and hydrophobic GelBond surfaces using the RNAeasy Plant Mini Kit (Qiagen Inc.) and used for reverse transcription. cDNA was prepared according to procedures for cDNA synthesis from total RNA in the Low RNA Input Fluorescent Linear Amplification Kit (Agilent Technologies, Inc.) with modification.

For qRT-PCR, total RNA was independently extracted from cAMP induced appressoria on the hydrophilic surface of GelBond as well as germinating spores without cAMP treatment, and cDNA was synthesized as described above. qRT-PCR reactions with *MPG1 *and *PTH11 *were carried out using 2× SYBR Green Master Mix (Applied Biosystems, Foster City, CA, USA) with that of the actin gene as an internal control. qRT-PCR was performed using the following profile: 94°C for 3 minutes; 30 cycles at 94°C, 45 s; 62°C, 45 s; 68°C, 2 minutes; and a final extension at 68°C for 7 minutes. Analysis of the qRT-PCR results was carried out as previously described by Livak and Schmittgen [126].

### Gene Ontology and functional annotation

Orthologs were identified between *M. oryzae *predicted proteins and proteins in the GO database [127] via searching reciprocal best hits with the following cut-offs; e-value, 1.0e-3, and identity, 20%. Results from local alignment using BLAST, functional domain comparisons from Interpro and prediction of signal peptides from SignalP 3.0 software and a manual literature review were used to make final assignments to GO functional categories.

### Targeted gene replacement and mutant screening

Gene replacement cassettes were constructed using adaptamer mediated PCR (Additional data file 5) [128]. Typically, 1.3 kb of upstream and downstream sequence of each target gene was amplified with primers that contained adaptamer sequences (Additional data file 6). A 1.5 kb fragment containing the hygromycin dehydrogenase gene with the *trp*C promoter from *A. nidulans *was amplified from plasmid PCB1003 using the adaptamer sequence attached to the forward HPHF and reverse HPHR primer set. Using nested primers (Additional data file 6) from inside of the 5' upstream fragment and from inside of the 3' end of the downstream fragment of the target gene, the individual fragments and hygromycin resistance gene fragment were combined and amplified together to construct a hygromycin cassette for gene replacement typically approximately 3.3 kb in length. The hygromycin cassette was transformed into 70-15 protoplasts as previously described [93]. Gene replacement mutants were identified by PCR screening (Additional data file 7) and further confirmed by Southern blot analysis.

### Mutant phenotype assays

A series of phenotype analyses were conducted on several knockout mutants (≥ 3) and ectopic (≥ 2) transformants for each gene functionally characterized. Germination and appressorium assays were conducted using spores collected from ten-day-old V8 agar plates and adjusted to 10^5 ^spores/ml. Spore suspension was spotted on the hydrophobic and hydrophilic surface of GelBond film and the rate of germination and appressorium formation was measured after 24 h incubation at 25°C in the dark. To test for pathogenicity, barley and rice seedlings were spray inoculated with *M. oryzae *spore suspension (3 × 10^4 ^spores/ml, Tween 20 0.025%) and incubated in dark humid conditions at 25°C. The number and size of lesions were recorded seven days post-inoculation. Growth rate assays were conducted by placing 10 μl of spore suspension (3 × 10^4 ^conidia/ml) on agar plates with complete or minimal media. In other growth rate assays, where minimal media was amended with glutamate and glutamine as a sole nitrogen source, glucose was also reduced to 0.125%. Colony morphology and diameters were recorded periodically for 15 days. The total number of spores on minimal media plates was counted after 15 days incubation. All experiments were conducted in triplicate and performed at least three times.

## Abbreviations

ABC, ATP-binding cassette; AI, appressorium initiation; AM, appressorium maturation; CI, cAMP-induced appressoria; cAMP, cyclic AMP; FKBP, FK506-binding protein; GO, Gene Ontology; MAPK, mitogen-activated protein kinase; NAD-GDH, NAD-specific glutamate dehydrogenase; PAL, phenylalanine ammonia-lyase; PKS, polyketide synthase; RT-PCR, reverse transcriptase PCR; SAGE, serial analysis of gene expression; SCD, synthalone dehydratase; THR, hydroxynaphthalene reductase; TOR, target of rapamycin.

## Authors' contributions

YYO, TM and RAD designed the study; YYO, ND and SC carried out the experiments; YYO and HP performed data processing and statistical analysis; DB and SM created the GO database; YYO, TM and RAD interpreted the data and wrote the manuscript.

## Additional data files

The following additional data are available with the online version of this paper. Additional data file 1 is a table listing GO categorization (biological process) of differentially expressed genes during spore germination. Additional data file 2 is a table listing primer sequences for RT-PCR and qRT-PCR. Additional data file 3 is a table listing categorization of appressorium consensus genes with known function. Additional data file 4 is a figure showing differential expression of the putative melanin biosynthesis gene cluster during appressorium formation. Additional data file 5 is a figure showing adaptamer mediated PCR strategy for targeted gene deletion. Additional data file 6 is a table listing primer sequences for gene specific replacement cassette construction. Additional data file 7 is a figure showing confirmation of target gene replacement by PCR.

## Supplementary Material

Additional data file 1 GO categorization (biological process) of differentially expressed genes during spore germination. Click here for file

Additional data file 2 Primer sequences for RT-PCR and qRT-PCR. Click here for file

Additional data file 3 Categorization of appressorium consensus genes with known function. Click here for file

Additional data file 4 Differential expression of the putative melanin biosynthesis gene cluster during appressorium formation. Click here for file

Additional data file 5 Adaptamer mediated PCR strategy for targeted gene deletion. Click here for file

Additional data file 6 Primer sequences for gene specific replacement cassette construction. Click here for file

Additional data file 7 Confirmation of target gene replacement by PCR. Click here for file
