# Supplementary material for: Transcriptome analysis reveals new insight into appressorium formation and function in the rice blast fungus Magnaporthe oryzae
Source: Genome Biol. 2008 May 20;9(5):R85. doi: 10.1186/gb-2008-9-5-r85 (PMC2441471; doi:10.1186/gb-2008-9-5-r85)
Supplement: Additional data file 3 — Categorization of appressorium consensus genes with known function. [file gb-2008-9-5-r85-S3.doc]

## **Additional** [**data file 3**](http://www.lib.ncsu.edu:2118/nature/journal/v434/n7036/suppinfo/nature03449.html)**. Categorization of appressorium consensus genes with known function**

| **Category** | **Gene ID** | **Exp.^a^** | **SignalP^b^** | **Blast hit** | **NCBI_ID** | **E-value^c^** |
| --- | --- | --- | --- | --- | --- | --- |
| **Melanin biosynthesis (GO:0042438)** | | | |  |  |  |
|  | MGG_07219 | UR | N | polyketide synthase [Colletotrichum lagenarium] | BAA18956.1 | 0 |
|  | MGG_07216 | UR | N | 1,3,6,8-trihydroxynaphthalene reductase [Magnaporthe grisea] | AAG29497.2 | 0 |
|  | MGG_05059 | UR | N | syctalone dehydratase I [Ceratocystis resinifera] | AAO60167.1 | 9.00E-73 |
|  | MGG_07218 | UR | N | Zn-II 2Cys6 regulatory protein [Leptosphaeria maculans] | AAO49457.1 | 1.00E-10 |
| **Secondary metabolism (GO:0019748)** | | | |  |  |  |
|  | MGG_00385 | UR | N | ochratoxin A non-ribosomal peptide synthetase [Penicillium nordicum] | AAS98174.1 | 6.00E-90 |
|  | MGG_00573 | UR | N | tetracenomycin polyketide synthesis O-methyltransferase [Mycobacterium smegmatis str. MC2 155] | ZP_00139515.1 | 5.00E-31 |
|  | MGG_00792 | UR | N | squalene-hopene-cyclase [Thermosynechococcus elongatus BP-1] | NP_683099.1 | E-112 |
|  | MGG_03397 | UR | N | Citrinin biosynthesis oxydoreductase CtnB [Monascus purpureus] | BAE95339.1 | 3.00E-63 |
|  | MGG_04335 | UR | N | nonribosomal peptide synthetase 12 [Cochliobolus heterostrophus] | AAX09994.1 | 9.00E-25 |
|  | MGG_04775 | UR | N | polyketide synthase [Botryotinia fuckeliana] | AAR90244.1 | 0 |
|  | MGG_04911 | UR | N | Fum15p [Gibberella moniliformis] | AAN74818.2 | 1.00E-56 |
|  | MGG_06585 | UR | N | Fum13p [Gibberella moniliformis] | AAN74816.1 | 1.00E-24 |
|  | MGG_10072 | UR | N | PKSN polyketide synthase for alternapyrone biosynthesis [Alternaria solani] | BAD83684.1 | 0 |
|  | MGG_01391 | DR | N | cytochrome P450 monooxygenase [Penicillium paxilli] | AAK11528.1 | 2.00E-52 |
| **Signal transduction (GO:0007165)** | | | |  |  |  |
|  | MGG_00871 | UR | N | Csr1p [Saccharomyces cerevisiae]. | NP_013484.1 | 6.00E-66 |
|  | MGG_00987 | UR | N | Plasma membrane protein involved in G-protein mediated pheromone [Saccharomyces cerevisiae] | NP_014226.1 | 6.00E-15 |
|  | MGG_01150 | UR | N | calcineurin temperature suppressor Cts1 [Cryptococcus neoformans] | AAN85205.1 | 7.00E-19 |
|  | MGG_01367 | UR | Y | secretory phospholipase A2 [Aspergillus oryzae] | BAD01582.1 | 1.00E-27 |
|  | MGG_05353 | UR | N | putative G protein-coupled receptor alpha [Botryotinia fuckeliana] | CAE55153.1 | 2.00E-16 |
|  | MGG_05804 | UR | N | phospholipase D [Emericella nidulans] | BAC67175.1 | 0 |
|  | MGG_08732 | UR | N | related to interferon-regulated resistance GTP-binding protein [Neurospora crassa] | CAE81930.1 | 0 |
|  | MGG_09570 | UR | Y | MAC1 interacting protein 1; ACI1 [Magnaporthe grisea] | AAN64312.1 | 2.00E-07 |
|  | MGG_09947 | UR | N | transmembrane protein, putative [Cryptococcus neoformans] | AAW42308.1 | 0 |
|  | MGG_01041 | DR | N | vivid PAS protein VVD [Neurospora crassa] | CAF06140.1 | 4.00E-45 |
|  | MGG_01094 | DR | N | conserved hypothetical protein [Neurospora crassa] | CAB91433.2 | 0 |
|  | MGG_02692 | DR | N | integral membrane protein [Magnaporthe grisea] | AAD30437.1 | 1.00E-05 |
|  | MGG_03148 | DR | N | bacterial signalling protein N terminal repeat family [Aspergillus fumigatus] | XP_753518.1 | 3.00E-86 |
|  | MGG_05214 | DR | Y | integral membrane protein [Magnaporthe grisea] | AAD30437.1 | 2.00E-24 |
|  | MGG_05871 | DR | Y | integral membrane protein, PTH11[Magnaporthe grisea] | AAD30436.1 | 0 |
|  | MGG_06035 | DR | N | probable peptidylprolyl isomerase (FK506-binding protein homolog) [Neurospora crassa] | CAF06078.1 | 2.00E-31 |
|  | MGG_10571 | DR | N | integral membrane protein [Magnaporthe grisea] | AAD30436.1 | 2.00E-17 |
|  | MGG_13736 | DR | N | putative G protein-coupled receptor alpha [Botryotinia fuckeliana] | CAE55153.1 | 2.00E-04 |
| **Amino Acid Metabolism (GO:0006520)** | | | |  |  |  |
|  | MGG_00189 | UR | N | delta-1-pyrroline-5-carboxylate dehydrogenase [Aspergillus fumigatus Af293] | XP_750764.1 | 0 |
|  | MGG_01906 | UR | N | putative nicotianamine synthase [Podospora anserina] | AAO25955.1 | 1.00E-30 |
|  | MGG_03231 | UR | N | related to pentachlorophenol 4-monooxygenase [Neurospora crassa] | CAD70786.1 | 7.00E-88 |
|  | MGG_05247 | UR | N | NAD(+)-specific glutamate dehydrogenase; NAD-GDH [Neurospora crassa] | AAB28355.1 | 0 |
|  | MGG_06095 | UR | N | cysteine dioxygenase [Ajellomyces capsulatus] | AAV66535.1 | 3.00E-52 |
|  | MGG_07224 | UR | N | threonine deaminase [Arxula adeninivorans] | CAA10977.1 | E-162 |
|  | MGG_09919 | UR | N | Amino transferase [Aspergillus fumigatus Af293] | XP_755696.1 | E-149 |
|  | MGG_10036 | UR | N | phenylalanine ammonia-lyase [Aspergillus fumigatus Af293] | XP_755245.1 | 2.00E-97 |
|  | MGG_10380 | UR | N | cystathionine-gamma-lyase [Acremonium chrysogenum] | AAF97598.1 | E-153 |
|  | MGG_10533 | UR | Y | arginase, putative [Cryptococcus neoformans var. neoformans JEC21] | AAW42854.1 | 3.00E-91 |
|  | MGG_02378 | DR | N | glutamate decarboxylase [Chaetomium globosum CBS 148.51] | EAQ84052.1 | 0 |
|  | MGG_02817 | DR | N | Glutamate decarboxylase [Saccharomyces cerevisiae] | NP_013976.1 | E-122 |
| **Proteolysis (GO:0006508)** | | | |  |  |  |
|  | MGG_00981 | UR | N | aspartyl proteinase [Trichoderma asperellum] | AAU11329.1 | 1.00E-79 |
|  | MGG_03056 | UR | Y | Aorsin [Aspergillus oryzae] | BAB97387.1 | E-132 |
|  | MGG_03260 | UR | N | related to calpain [Neurospora crassa] | CAD37034.1 | E-166 |
|  | MGG_03580 | UR | N | Atg4p [Saccharomyces cerevisiae] | NP_014176.2 | 1.00E-49 |
|  | MGG_03670 | UR | Y | subtilisin-like serine protease [Podospora anserina] | AAC03564.2 | 0 |
|  | MGG_07404 | UR | Y | tripeptidyl aminopeptidase [Aspergillus oryzae] | AAU10333.1 | E-126 |
|  | MGG_08526 | UR | N | thiol protease [Porphyromonas gingivalis] | AAA25652.1 | 2.00E-17 |
|  | MGG_09246 | UR | Y | subtilisin-like protease [Verticillium dahliae] | AAR10770.1 | 7.00E-82 |
|  | MGG_09351 | UR | Y | aspartyl protease [Sclerotinia sclerotiorum] | AAF76202.1 | E-126 |
|  | MGG_09716 | UR | Y | carboxypeptidase [Metarhizium anisopliae] | AAB68600.1 | E-119 |
|  | MGG_09032 | DR | Y | related to acid proteinase [Neurospora crassa] | CAD36982.1 | 1.00E-18 |
| **Carbohydrate Metabolism (GO:0005975)** | | | |  |  |  |
|  | MGG_03880 | UR | N | alcohol dehydrogenase [Cochliobolus lunatus] | ABC88428.1 | E-138 |
|  | MGG_05908 | UR | Y | ALK1 [Yarrowia lipolytica] | BAA31433.1 | 3.00E-86 |
|  | MGG_00695 | UR | N | alpha-1,2-mannosidase subfamily [Aspergillus fumigatus Af293] | XP_751252.1 | 0 |
|  | MGG_05246 | UR | Y | alpha-L-rhamnosidase A precursor [Aspergillus aculeatus] | AAK16249.1 | 3.00E-28 |
|  | MGG_00994 | UR | Y | alpha-mannosidase [Aspergillus saitoi] | BAA08634.1 | E-149 |
|  | MGG_00659 | UR | Y | beta-1,3-exoglucanase [Trichoderma hamatum] | AAP33112.1 | 0 |
|  | MGG_10494 | UR | Y | beta-1,4-mannosyltransferase, putative [Cryptococcus neoformans] | AAW41187.1 | 3.00E-72 |
|  | MGG_10051 | UR | Y | cellobiose dehydrogenase [Aspergillus fumigatus Af293] | XP_747382.1 | 3.00E-13 |
|  | MGG_00086 | UR | Y | chitinase [Trichoderma viride] | AAG09447.1 | E-145 |
|  | MGG_01876 | UR | Y | chitinase 3 [Coccidioides posadasii] | AAO88269.1 | 2.00E-60 |
|  | MGG_03307 | UR | N | chitinase, class I [Myxococcus xanthus DK 1622] | YP_635494.1 | 2.00E-03 |
|  | MGG_02393 | UR | Y | cutinase [Botryotinia fuckeliana] | CAA93255.1 | 6.00E-38 |
|  | MGG_11966 | UR | Y | cutinase [Botryotinia fuckeliana] | CAA93255.1 | 3.00E-48 |
|  | MGG_05735 | UR | N | FMN dependent dehydrogenase [Aspergillus fumigatus Af293] | XP_747805.1 | E-109 |
|  | MGG_10878 | UR | N | GAOA_DACDE Galactose oxidase precursor (GAO) [Gibberella zeae PH-1] | XP_391208.1 | E-107 |
|  | MGG_03361 | UR | N | glycosyl transferase [Aspergillus fumigatus Af293] | XP_751278.1 | E-126 |
|  | MGG_01231 | UR | N | L-arabinitol 4-dehydrogenase [Hypocrea jecorina] | AAP57209.1 | E-161 |
|  | MGG_02813 | UR | N | malate synthase [Candida tropicalis] | BAA02681.1 | 0 |
|  | MGG_00450 | UR | N | phosphoenolpyruvate carboxykinase [Emericella nidulans] | AAL10705.1 | 0 |
|  | MGG_01922 | UR | N | polysaccharide deacetylase family protein [Pseudomonas syringae] | YP_276070.1 | E-110 |
|  | MGG_00625 | UR | N | probable glucosamine-6-phosphate deaminase [Neurospora crassa] | CAE85549.1 | E-103 |
|  | MGG_00077 | UR | N | proteophosphoglycan ppg4 [Leishmania major strain Friedlin] | AAZ14280.1 | 1.00E-05 |
|  | MGG_10038 | UR | Y | putative beta-glucosidase [Arthrobacter nicotinovorans] | CAD47965.1 | E-126 |
|  | MGG_03497 | UR | N | pyruvate dehydrogenase [Aedes aegypti] | EAT32992.1 | 9.00E-01 |
|  | MGG_03263 | UR | N | related to aldehyde dehydrogenase (NAD+) [Neurospora crassa] | CAD37029.1 | E-152 |
|  | MGG_00623 | UR | N | related to hexokinase [Neurospora crassa] | CAE85550.1 | E-124 |
|  | MGG_00316 | DR | Y | a-L-fucosidase [Halocynthia roretzi] | BAB85519.1 | 4.00E-53 |
|  | MGG_05364 | DR | Y | endoglucanase, putative [Aspergillus fumigatus] | CAF31975.1 | 1.00E-57 |
|  | MGG_05785 | DR | Y | Fructosyltransferase [Aspergillus sydowii] | CAB89083.1 | 8.00E-47 |
|  | MGG_01096 | DR | N | glucan 1, 4-alpha-glucosidase [Neurospora crassa] | CAB91426.1 | 0 |
|  | MGG_03041 | DR | N | glucokinase [Aspergillus niger] | CAA67949.1 | E-138 |
|  | MGG_10400 | DR | Y | glucosidase [Fusarium sporotrichioides] | AAO27749.1 | 8.00E-25 |
|  | MGG_10005 | DR | N | glycerol kinase [Aspergillus fumigatus Af293] | XP_750736.1 | E-167 |
|  | MGG_13485 | DR | N | probable 2-isopropylmalalate synthase [Neurospora crassa] | CAE76195.1 | 0 |
|  | MGG_09238 | DR | N | rhamnose biosynthetic enzyme 1, putative, expressed [Oryza sativa | ABF95279.1 | 2.00E-79 |
|  | MGG_01320 | DR | N | UDP-N-acetylglucosamine pyrophosphorylase [Emericella nidulans] | AAW49004.1 | 0 |
| **Lipid metabolism (GO:0006629)** | | | |  |  |  |
|  | MGG_11040 | UR | Y | 1-acyl-sn-gylcerol-3-phosphate acyltransferase [Saccharomyces cerevisiae] | NP_010231.1 | 8.00E-44 |
|  | MGG_03765 | UR | Y | 7-dehydrocholesterol reductase [Homo sapiens] | NP_001351.1 | 7.00E-93 |
|  | MGG_07613 | UR | N | cut6 [Schizosaccharomyces pombe] | CAB16395.1 | 0 |
|  | MGG_08257 | UR | N | fatty acid transporter protein [Cochliobolus heterostrophus] | CAA75802.1 | 2.00E-96 |
|  | MGG_06704 | UR | N | Gde1p [Saccharomyces cerevisiae] | NP_015215.1 | 0 |
|  | MGG_01026 | UR | N | hydroxymethylglutaryl-CoA synthase [Aspergillus fumigatus Af293] | XP_754553.1 | 0 |
|  | MGG_11317 | UR | N | long chain fatty alcohol oxidase [Aspergillus fumigatus Af293] | XP_753079.1 | E-145 |
|  | MGG_00853 | UR | N | oxysterol binding protein-like 11 [Mus musculus] | NP_789810.1 | 8.00E-24 |
|  | MGG_07337 | UR | N | probable peroxisomal protein POX18 [Neurospora crassa] | CAD21491.1 | 2.00E-41 |
|  | MGG_03569 | UR | N | putative delta 8-sphingolipid desaturase [Kluyveromyces lactis] | BAB93118.1 | E-143 |
|  | MGG_03690 | UR | N | related to ethanolaminephosphotransferase [Neurospora crassa] | CAE76360.1 | 4.00E-61 |
|  | MGG_02409 | UR | N | sterol carrier protein [Aspergillus fumigatus Af293] | XP_752186.1 | 0 |
|  | MGG_10879 | DR | N | fatty acid omega-hydroxylase (P450foxy) [Fusarium oxysporum] | BAA82526.1 | 0 |
|  | MGG_01925 | DR | N | fatty acid omega-hydroxylase (P450foxy) [Fusarium oxysporum] | BAA82526.1 | 0 |
|  | MGG_02610 | DR | N | lipase [Bacillus cereus ATCC 10987] | NP_979556.1 | 1.00E-20 |
| **Cell development (GO:0007275)** | | | |  |  |  |
|  | MGG_06461 | UR | N | PREDICTED: similar to G2/mitotic-specific cyclin F [Strongylocentrotus purpuratus] | XP_798496.1 | 5.00E-09 |
|  | MGG_00850 | UR | N | SesB [Nectria haematococca] | AAS80314.1 | 4.00E-27 |
|  | MGG_06538 | DR | Y | blastomyces yeast phase-specific protein 1 [Ajellomyces dermatitidis] | AAF86474.1 | 2.00E-07 |
|  | MGG_00513 | DR | N | con-8 protein [Neurospora crassa] | CAE76281.1 | 2.00E-06 |
|  | MGG_03336 | DR | N | late embryogenesis abundant protein [Catharanthus roseus] | AAY84145.1 | 9.00E-05 |
| **Cell wall & surface (NA)** | | | |  |  |  |
|  | MGG_02796 | UR | N | cell surface flocculin [Saccharomyces cerevisiae] | CAK18547.1 | 1.00E-03 |
|  | MGG_03436 | UR | N | cell wall mannoprotein MnpA [Aspergillus nidulans] | AAM16156.1 | 1.00E-03 |
|  | MGG_09460 | UR | Y | cell wall protein [Aspergillus kawachii] | BAD01559.1 | 2.00E-18 |
|  | MGG_02778 | UR | Y | cell wall protein [Aspergillus kawachii] | BAD01559.1 | 2.00E-03 |
|  | MGG_04891 | UR | N | GPI-anchored cell surface glycoprotein (flocculin) [Saccharomyces revisiae] | NP_012284.1 | 1.00E-02 |
|  | MGG_09477 | UR | N | related to a-agglutinin core protein AGA1 [Neurospora crassa] | CAC28825.2 | 2.00E-13 |
|  | MGG_04093 | DR | N | cell wall surface anchor family protein [Streptococcus pneumoniae TIGR4] | AAK75846.1 | 1.00E-38 |
|  | MGG_04913 | DR | N | cyanovirin-N-like protein [Tuber borchii] | AAV85993.1 | 2.00E-05 |
| **Electron transport (GO:0006118)** | | | |  |  |  |
|  | MGG_01473 | UR | N | 2-nitropropane dioxygenase, putative [Cryptococcus neoformans] | AAW45147.1 | E-102 |
|  | MGG_13573 | UR | N | 6-hydroxy-D-nicotine oxidase [Coccidioides immitis RS] | EAS36048.1 | 9.00E-50 |
|  | MGG_09162 | UR | Y | cytochrome b2, mitochondrial precursor [Cryptococcus neoformans ] | AAW45006.1 | 6.00E-50 |
|  | MGG_04774 | UR | N | Fsh3p [Saccharomyces cerevisiae] | NP_014923.1 | 2.00E-05 |
|  | MGG_11608 | UR | Y | laccase [Gaeumannomyces graminis var. tritici] | CAD10747.1 | 0 |
|  | MGG_07790 | UR | Y | manganese peroxidase [Ganoderma applanatum] | BAA88392.1 | 3.00E-24 |
|  | MGG_09453 | UR | Y | monooxygenase [Aspergillus flavus] | AAT65719.1 | 2.00E-24 |
|  | MGG_02256 | UR | N | monooxygenase [Penicillium paxilli] | AAK11530.1 | 4.00E-41 |
|  | MGG_00790 | UR | N | oxidoreductase [Agrobacterium tumefaciens str. C58] | NP_533225.1 | 5.00E-07 |
|  | MGG_07793 | UR | N | oxidoreductase, short-chain dehydrogenase/reductase family[Aspergillus fumigatus Af293] | XP_749588.1 | 1.00E-40 |
|  | MGG_13764 | UR | N | polyphenol oxidase [Acremonium murorum] | CAB75422.1 | 0 |
|  | MGG_02792 | UR | Y | related to n-alkane-inducible cytochrome P450 [Neurospora crassa] | CAC10088.1 | E-116 |
|  | MGG_01569 | DR | N | probable 1, 4-Benzoquinone reductase [Neurospora crassa] | CAE76242.1 | 2.00E-71 |
|  | MGG_04120 | DR | N | putative monooxygenase [Bradyrhizobium japonicum USDA 110] | NP_770497.1 | E-109 |
|  | MGG_02798 | DR | N | Rossman fold oxidoreductase, putative [Cryptococcus neoformans] | AAW42118.1 | 2.00E-31 |
|  | MGG_10800 | DR | N | sarcosine oxidase [Cylindrocarpon didymum] | BAA96069.1 | E-168 |
|  | MGG_00357 | DR | N | Short-chain dehydrogenase/reductase SDR [Mycobacterium sp. JLS] | ZP_01278292.1 | 1.00E-18 |
| **Metabolism (GO:0008152)** | | | |  |  |  |
|  | MGG_09942 | UR | Y | alpha/beta hydrolase [Aspergillus fumigatus Af293] | XP_750599.1 | 5.00E-55 |
|  | MGG_02497 | UR | Y | carboxylesterase [Aspergillus fumigatus Af293] | XP_753283.1 | 1.00E-48 |
|  | MGG_00745 | UR | N | hydrolase [Aspergillus fumigatus Af293] | XP_753376.1 | 7.00E-47 |
|  | MGG_12475 | UR | N | phloretin hydrolase [Eubacterium ramulus] | AAQ12341.1 | 6.00E-17 |
|  | MGG_06823 | UR | Y | putative purple acid phosphatase [Arabidopsis thaliana] | AAT48877.1 | 2.00E-41 |
|  | MGG_04378 | DR | N | Alpha/beta hydrolase [Burkholderia sp. 383] | ABB10796.1 | 4.00E-09 |
|  | MGG_02987 | DR | N | Carboxylesterase, type B [Mycobacterium flavescens PYR-GCK] | ZP_01191056.1 | 4.00E-37 |
|  | MGG_06911 | DR | N | probable inosine triphosphate pyrophosphatase [Neurospora crassa] | CAD70978.1 | 3.00E-87 |
| **Protein modification (GO:0006464)** | | | |  |  |  |
|  | MGG_01282 | UR | N | Ubi4p [Saccharomyces cerevisiae] | NP_013061.1 | E-166 |
|  | MGG_07297 | UR | N | ubiquitin-like conjugating enzyme, putative [Cryptococcus neoformans] | AAW45433.1 | E-138 |
| **Protein biosynthesis (GO:0006412)** | | | |  |  |  |
|  | MGG_06479 | DR | N | 40S ribosomal protein S22 [Coccidioides immitis RS] | EAS32089.1 | 2.00E-63 |
|  | MGG_05449 | DR | N | 60S ribosomal protein L16 [Chaetomium globosum CBS 148.51] | EAQ86180.1 | E-102 |
|  | MGG_04921 | DR | N | 60S ribosomal protein L23 [Chaetomium globosum CBS 148.51] | EAQ93853.1 | 3.00E-73 |
|  | MGG_06721 | DR | N | 60S ribosomal protein L28 [Chaetomium globosum CBS 148.51] | EAQ86004.1 | 5.00E-61 |
|  | MGG_03193 | DR | N | 60S ribosomal protein L3 [Chaetomium globosum CBS 148.51] | EAQ83793.1 | 0 |
|  | MGG_07048 | DR | N | 60S RIBOSOMAL PROTEIN L5 [Neurospora crassa] | CAD71058.1 | E-133 |
|  | MGG_04829 | DR | N | 60S ribosomal protein L9 b [Aspergillus fumigatus Af293] | XP_752277.1 | 2.00E-81 |
|  | MGG_06269 | DR | N | ADL127Cp [Ashbya gossypii ATCC 10895] | AAS51793.1 | E-118 |
|  | MGG_07753 | DR | N | cytosolic large ribosomal subunit L11 [Aspergillus fumigatus Af293] | XP_752052.1 | 2.00E-80 |
|  | MGG_10680 | DR | N | probable 40S RIBOSOMAL PROTEIN S24 [Neurospora crassa] | CAD71100.1 | 1.00E-48 |
|  | MGG_04455 | DR | N | probable ribosomal protein L35 [Neurospora crassa] | CAE76503.1 | 7.00E-51 |
|  | MGG_06952 | DR | N | probable ribosomal protein L38 [Neurospora crassa] | CAC28690.1 | 6.00E-19 |
|  | MGG_04612 | DR | N | probable ribosomal protein L7a.e.B, cytosolic [Neurospora crassa] | CAE85573.1 | 6.00E-97 |
|  | MGG_04484 | DR | N | Protein component of the large (60S) ribosomal subunit [Saccharomyces cerevisiae] | NP_014521.1 | 4.00E-47 |
|  | MGG_03727 | DR | N | putative 60s ribosomal protein [Colletotrichum gloeosporioides]. | CAC15500.1 | 2.00E-48 |
|  | MGG_02659 | DR | N | ribosomal protein L14 [Aspergillus fumigatus Af293] | XP_747694.1 | 2.00E-38 |
|  | MGG_05296 | DR | N | ribosomal protein L34-like protein [Ophiostoma novo-ulmi] | AAK58051.1 | 2.00E-53 |
|  | MGG_05716 | DR | Y | ribosomal protein S1 [Geobacter metallireducens GS-15] | ABB31109.1 | 1.00E+00 |
|  | MGG_02953 | DR | N | ribosomal protein Srp1 [Sclerotinia sclerotiorum] | AAP58401.1 | 2.00E-34 |
|  | MGG_03554 | DR | N | RL36_TRIHM 60S ribosomal protein L36 (TRP36) [Gibberella zeae PH-1] | XP_381414.1 | 4.00E-34 |
|  | MGG_06480 | DR | N | RS12_ERYGR 40S ribosomal protein S12 [Gibberella zeae PH-1] | XP_387468.1 | 8.00E-64 |
|  | MGG_06837 | DR | N | RS21_NEUCR 40S ribosomal protein S21 (CRP7) [Gibberella zeae | XP_380974.1 | 8.00E-38 |
| **Response to stress (GO:0006950)** | | | |  |  |  |
|  | MGG_06747 | UR | N | glutathione S-transferase [Botryotinia fuckeliana] | AAG43132.1 | 5.00E-96 |
|  | MGG_04358 | UR | N | hsp16 [Schizosaccharomyces pombe] | CAA19006.1 | 5.00E-06 |
|  | MGG_03350 | UR | Y | related to cytosolic Cu/Zn superoxide dismutase [Neurospora crassa] | CAB97297.1 | 4.00E-25 |
|  | MGG_10061 | DR | N | catalase [Cochliobolus heterostrophus] | AAR17473.1 | 0 |
|  | MGG_03896 | DR | N | chloride peroxidase [Burkholderia cepacia] | AAL73575.1 | 4.00E-04 |
| **Regulation of transcription (GO:0006355)** | | | |  |  |  |
|  | MGG_00320 | UR | N | C6 transcription factor [Aspergillus fumigatus Af293] | XP_749091.1 | 2.00E-05 |
|  | MGG_08295 | UR | N | C6 transcription factor [Aspergillus fumigatus Af293] | XP_750890.1 | 2.00E-20 |
|  | MGG_03288 | UR | N | CAMP responsive element binding protein 3-like 2 [Mus musculus] | AAH43466.1 | 1.00E-04 |
|  | MGG_09276 | UR | N | cercosporin resistance protein [Cercospora nicotianae] | AAD25072.2 | 4.00E-31 |
|  | MGG_06539 | UR | N | CipA protein, putative [Aspergillus fumigatus] | CAE47970.1 | 5.00E-44 |
|  | MGG_02129 | UR | N | regulatory protein AlcR [Aspergillus fumigatus Af293] | XP_748311.1 | E-110 |
|  | MGG_09031 | DR | N | sporulation negative regulatory protein-like protein [Magnaporthe grisea] | AAX07687.1 | E-131 |
|  | MGG_06832 | DR | N | transcription activator-like protein [Nectria haematococca] | AAO72069.1 | 1.00E-03 |
|  | MGG_01490 | DR | N | Transcriptional Regulator, AraC family [Pseudomonas fluorescens PfO-1] | ABA73699.1 | 7.00E-05 |
|  | MGG_09200 | DR | N | zinc finger protein [Ascobolus immersus] | CAA67549.1 | 3.00E-65 |
| **Transport (GO:0006810)** | | | |  |  |  |
|  | MGG_10410 | UR | N | ABC transporter [Aspergillus fumigatus Af293] | XP_754651.1 | 0 |
|  | MGG_02167 | UR | N | aflatoxin efflux pump [Aspergillus flavus] | AAM53947.1 | E-113 |
|  | MGG_04105 | UR | Y | cation transport-related protein, putative [Cryptococcus neoformans] | AAW42114.1 | 3.00E-22 |
|  | MGG_00930 | UR | N | copper resistance-associated P-type ATPase [Candida albicans] | AAF04593.1 | E-177 |
|  | MGG_04068 | UR | N | fructose symporter [Aspergillus fumigatus Af293] | XP_747651.1 | 3.00E-11 |
|  | MGG_06336 | UR | Y | integral membrane protein [Aspergillus fumigatus Af293] | XP_750355.1 | E-102 |
|  | MGG_09827 | UR | N | Jen1p [Saccharomyces cerevisiae] | NP_012705.1 | 2.00E-90 |
|  | MGG_02763 | UR | N | Lpe10p [Saccharomyces cerevisiae] | NP_015265.1 | 2.00E-58 |
|  | MGG_12650 | UR | N | MFS transporter [Aspergillus fumigatus Af293] | XP_754894.1 | 1.00E-74 |
|  | MGG_13192 | UR | N | MFS transporter [Aspergillus fumigatus Af293] | XP_747222.1 | E-122 |
|  | MGG_04511 | UR | N | MFS transporter [Beauveria bassiana] | AAO73599.1 | 1.00E-82 |
|  | MGG_00275 | UR | N | MFS transporter [Beauveria bassiana] | AAO73599.1 | 2.00E-88 |
|  | MGG_11717 | UR | N | MFS_transporter protein [Streptomyces avermitilis] | BAB69375.1 | 1.00E-14 |
|  | MGG_03640 | UR | N | Mfs1.1 [Coprinus cinereus] | AAF01426.1 | 4.00E-69 |
|  | MGG_03843 | UR | N | multidrug transporter, putative [Cryptococcus neoformans] | AAW45439.1 | 2.00E-72 |
|  | MGG_07228 | UR | N | peptide transporter MTD1 [Schizophyllum commune] | AAF26618.1 | 5.00E-77 |
|  | MGG_03957 | UR | N | Permease of the drug/metabolite transporter, DMT superfamily [Prochlorococcus marinus str. MIT 9211] | ZP_01005589.1 | 4.00E-12 |
|  | MGG_04994 | UR | N | plasma membrane H+-ATPase [Blumeria graminis] | AAK94188.1 | 0 |
|  | MGG_07494 | UR | N | possible cation efflux protein [Aspergillus fumigatus] | CAF32159.1 | E-129 |
|  | MGG_02124 | UR | N | potassium transporter hak-1 [Neurospora crassa] | CAE81927.1 | 0 |
|  | MGG_01778 | UR | N | probable aflatoxin efflux pump AFLT [Neurospora crassa] | CAF06057.1 | E-163 |
|  | MGG_10869 | UR | N | probable aflatoxin efflux pump AFLT [Neurospora crassa] | CAF06057.1 | 3.00E-21 |
|  | MGG_08258 | UR | N | probable PEPTIDE TRANSPORTER PTR2 [Neurospora crassa] | CAE75697.1 | 0 |
|  | MGG_04852 | UR | N | probable P-type ATPase [Neurospora crassa] | CAE76097.1 | 0 |
|  | MGG_05085 | UR | N | putative ion transporter [Candida albicans SC5314] | XP_712877.1 | 3.00E-71 |
|  | MGG_06794 | UR | N | related to aminotriazole resistance protein [Neurospora crassa] | CAF06028.1 | E-173 |
|  | MGG_07062 | UR | N | related to monocarboxylate transporter [Neurospora crassa] | CAD70416.1 | 3.00E-25 |
|  | MGG_10200 | UR | N | small oligopeptide transporter, OPT family [Aspergillus fumigatus] | XP_755859.1 | 0 |
|  | MGG_06604 | UR | Y | Vacuolar membrane protein involved in the ATP-dependent transport [Saccharomyces cerevisiae] | NP_012476.1 | 4.00E-56 |
|  | MGG_08968 | DR | N | allantoate transporter, putative [Cryptococcus neoformans] | AAW44174.1 | 1.00E-79 |
|  | MGG_09193 | DR | N | l-fucose permease [Aspergillus fumigatus Af293] | XP_750566.1 | E-148 |
|  | MGG_03298 | DR | N | phthalate transporter [Aspergillus fumigatus Af293] | XP_746562.1 | E-132 |
|  | MGG_01480 | DR | N | related to large-conductance mechanosensitive channel [Neurospora crassa] | CAB91331.2 | 1.00E-36 |
|  | MGG_07843 | DR | N | Tna1 [Paracoccidioides brasiliensis] | AAQ04627.1 | E-152 |
|  | MGG_04927 | DR | N | transporter-like protein [Magnaporthe grisea] | AAX07640.1 | 0 |
| **Pathogenecity (GO:0009405)** | | | |  |  |  |
|  | MGG_04202 | UR | Y | putative Egh16H1 precursor isoform B [Blumeria graminis] | AAK25793.1 | E-107 |
|  | MGG_02253 | UR | Y | putative Egh16H1 precursor isoform B [Blumeria graminis] | AAK25793.1 | 3.00E-38 |
|  | MGG_00438 | UR | Y | pathogenicity protein [Magnaporthe grisea] | AAD01641.1 | E-121 |
|  | MGG_12337 | UR | Y | ASG1 [Magnaporthe grisea] | AAL28112.1 | 2.00E-75 |
|  | MGG_09875 | DR | Y | ASG1 [Magnaporthe grisea] | AAL28112.1 | 1.00E-72 |
|  | MGG_10315 | DR | N | MPG1[Magnaporthe grisea 70-15] | XP_366095.1 | 9.00E-42 |
|  | MGG_05344 | DR | Y | snodprot-FS [Gibberella pulicaris] | AAV83793.1 | 3.00E-44 |
| **Others** | | | |  |  |  |
|  | MGG_06847 | UR | N | annexin XIV [Neurospora crassa] | CAF06024.1 | E-112 |
|  | MGG_04726 | UR | N | DNA repair system specific for alkylated DNA [Xanthomonas oryzae] | YP_199441.1 | 3.00E-08 |
|  | MGG_05528 | UR | N | probable cytosceletal binding protein [Neurospora crassa] | CAD21322.1 | E-136 |
|  | MGG_04740 | UR | N | putative SMK toxin resistance protein [Candida albicans SC5314] | XP_713262.1 | 7.00E-06 |
|  | MGG_04110 | UR | N | queuine tRNA-ribosyltransferase [Aspergillus fumigatus Af293] | XP_746611.1 | E-161 |
|  | MGG_09096 | UR | N | related to tol protein [Neurospora crassa] | CAD70524.1 | 9.00E-34 |
|  | MGG_05053 | UR | N | UbiE/COQ5 methyltransferase [Aspergillus fumigatus Af293] | XP_748902.1 | 1.00E-26 |
|  | MGG_08008 | DR | N | formyltetrahydrofolate deformylase [Aspergillus fumigatus Af293] | XP_751045.1 | 1.00E-93 |
|  | MGG_07749 | DR | Y | putative alpha subunit of ATP synthase [Cyathea arborea] | CAJ44964.1 | 2.00E-04 |
|  | MGG_03270 | DR | N | putative U3 snoRNP component [Candida albicans SC5314] | XP_722611.1 | E-113 |
|  | MGG_09461 | DR | N | UV-endonuclease UVE-1 [Neurospora crassa] | CAD21267.1 | E-178 |

^a^ Gene expression marked as upregulated (UR) or down regulated(DR).

^b^ Signal peptide cleavage sites predicted by SignalP 3.0 ([www.cbs.dtu.dk/services/SignalP/](http://www.cbs.dtu.dk/services/SignalP/))

^c^ E-values taken from BLASTX search against NCBI non redundant protein database.
