# Supplementary material for: Transcriptome analysis reveals new insight into appressorium formation and function in the rice blast fungus Magnaporthe oryzae
Source: Genome Biol. 2008 May 20;9(5):R85. doi: 10.1186/gb-2008-9-5-r85 (PMC2441471; doi:10.1186/gb-2008-9-5-r85)
Supplement: Additional data file 4 — Differential expression of the putative melanin biosynthesis gene cluster during appressorium formation. [file gb-2008-9-5-r85-S4.doc]

## **Additional data file 4. Differential expression of the putative melanin biosynthesis gene cluster during appressorium formation**
