# Supplementary material for: Transcriptome analysis reveals new insight into appressorium formation and function in the rice blast fungus Magnaporthe oryzae
Source: Genome Biol. 2008 May 20;9(5):R85. doi: 10.1186/gb-2008-9-5-r85 (PMC2441471; doi:10.1186/gb-2008-9-5-r85)

## [**Additional data file 5**](http://www.lib.ncsu.edu:2118/nature/journal/v434/n7036/suppinfo/nature03449.html)**. Adaptamer mediated PCR strategy for targeted gene deletion.**


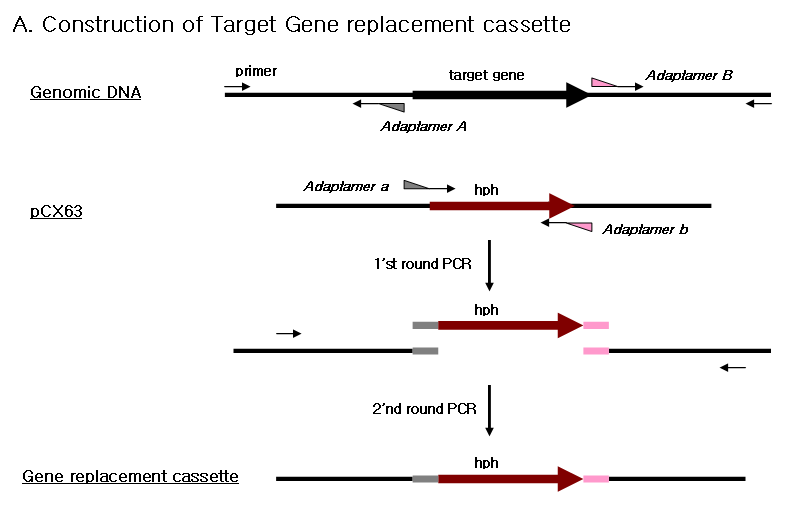


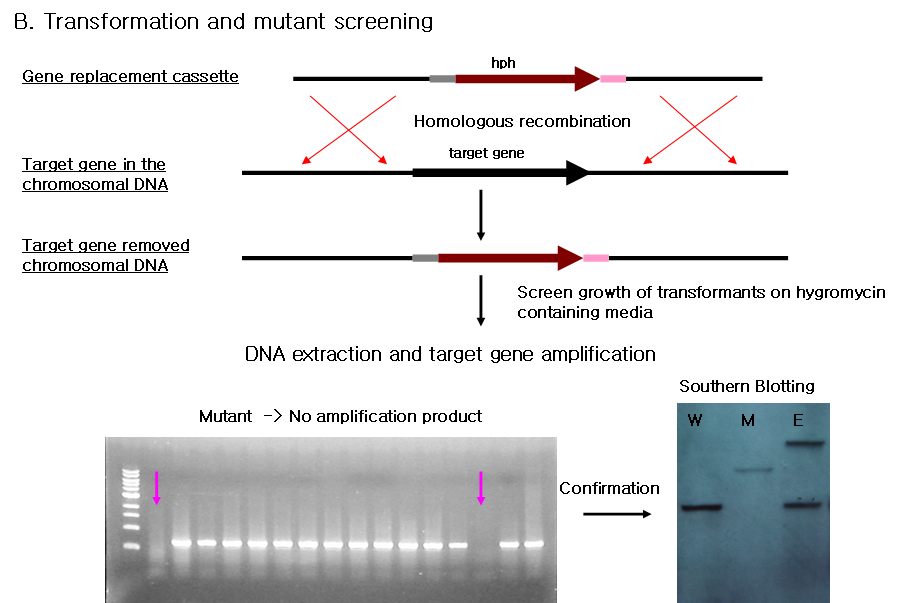

Supplement: Additional data file 5 — Adaptamer mediated PCR strategy for targeted gene deletion. [file gb-2008-9-5-r85-S5.doc]
