# Supplementary material for: Transcriptome analysis reveals new insight into appressorium formation and function in the rice blast fungus Magnaporthe oryzae
Source: Genome Biol. 2008 May 20;9(5):R85. doi: 10.1186/gb-2008-9-5-r85 (PMC2441471; doi:10.1186/gb-2008-9-5-r85)

## [**Additional data file 7**](http://www.lib.ncsu.edu:2118/nature/journal/v434/n7036/suppinfo/nature03449.html)**. Confirmation of target gene replacement by PCR.**

The correct replacement of target gene with hygromycin dehydrogenase gene in mutants (M1, M2) was verified by the absence of PCR products of the expected size using target gene specific primers (A) as well as by the appearance of expected PCR products generated from a primer outside of the left (B) or right (C) border flanking sequences of the target gene used in the gene replacement cassette and hygromycin dehydrogenase gene specific primers. Presence of the hygromycin dehydrogenase gene in mutant and ectopic (E1, E2) strains was confirmed by the presence of a PCR product using hygromycin dehydrogenase gene specific primer pairs (D) which was absent in wild type 70-15 (W).

Similar PCR results were obtained from all mutant and ectopic strains generated in this study.


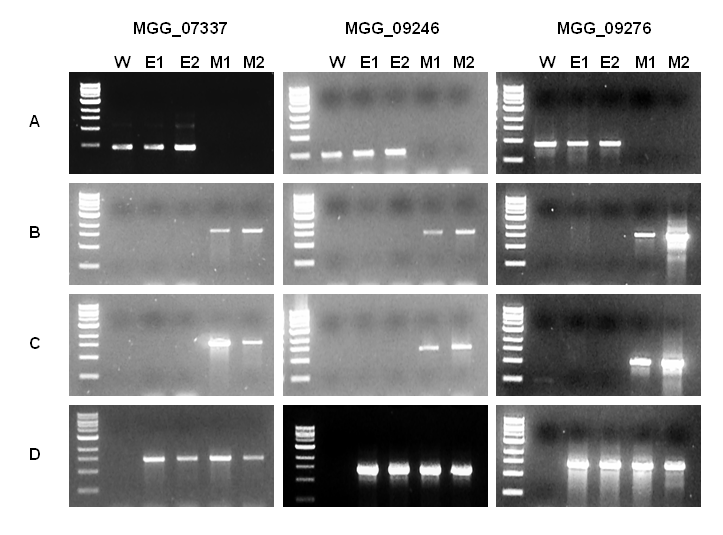

Supplement: Additional data file 7 — Confirmation of target gene replacement by PCR. [file gb-2008-9-5-r85-S7.doc]
